# A linker protein from a red-type pyrenoid phase separates with Rubisco via oligomerizing sticker motifs

**DOI:** 10.1073/pnas.2304833120

**Published:** 2023-06-13

**Authors:** Zhen Guo Oh, Warren Shou Leong Ang, Cheng Wei Poh, Soak-Kuan Lai, Siu Kwan Sze, Hoi-Yeung Li, Shashi Bhushan, Tobias Wunder, Oliver Mueller-Cajar

**Affiliations:** ^a^School of Biological Sciences, Nanyang Technological University, Singapore 637551, Singapore; ^b^Nanyang Institute of Structural Biology, Nanyang Technological University, Singapore 639798, Singapore

**Keywords:** Rubisco, pyrenoid, CO_2_ fixation, phase separation, diatoms

## Abstract

The slow kinetics and poor substrate specificity of the key photosynthetic CO_2_-fixing enzyme Rubisco have prompted the repeated evolution of Rubisco-containing biomolecular condensates known as pyrenoids in the majority of eukaryotic microalgae. Diatoms dominate marine photosynthesis, but the interactions underlying their pyrenoids are unknown. Here, we identify and characterize the Rubisco linker protein PYCO1 from *Phaeodactylum tricornutum*. PYCO1 is a tandem repeat protein containing prion-like domains that localizes to the pyrenoid. It undergoes homotypic liquid–liquid phase separation (LLPS) to form condensates that specifically partition diatom Rubisco. Saturation of PYCO1 condensates with Rubisco greatly reduces the mobility of droplet components. Cryo–electron microscopy and mutagenesis data revealed the sticker motifs required for homotypic and heterotypic phase separation. Our data indicate that the PYCO1–Rubisco network is cross-linked by PYCO1 stickers that oligomerize to bind to the small subunits lining the central solvent channel of the Rubisco holoenzyme. A second sticker motif binds to the large subunit. Pyrenoidal Rubisco condensates are highly diverse and tractable models of functional LLPS.

## Significance

Diatoms are mostly marine algae responsible for up to 20% of global carbon dioxide fixation. To overcome the slow and nonspecific properties of the CO_2_-fixing enzyme Rubisco, it is sequestered in a subcellular compartment called the pyrenoid, allowing CO_2_ gas to be concentrated there. We discovered the repeat protein PYCO1, which can phase separate to form dense protein droplets in the test tube. The droplets specifically bind large quantities of diatom Rubisco resulting in much larger and less dynamic droplets. This phenomenon is caused by repeating elements on PYCO1 that oligomerize and bind to the Rubisco small subunits. Pyrenoids are ubiquitous and derived by convergent evolution. Understanding their formation provides a framework to introduce them into plants.

Almost all biological CO_2_ fixation is catalyzed by ribulose 1,5-bisphosphate carboxylase/oxygenase (Rubisco), and about half of this activity is performed by marine photoautotrophs (1). Most marine unicellular algae have compensated for Rubisco’s catalytic shortcomings by evolving CO_2_ concentrating mechanisms, which utilize active transport to concentrate the abundant bicarbonate ion in the chloroplast stroma (2–4). Strategically placed carbonic anhydrases then catalyze the formation of CO_2_ gas in the vicinity of Rubisco active sites. To achieve significant local elevation of CO_2_ gas concentrations, the volume occupied by Rubisco needs to be minimized, which has led to the convergent evolution of the pyrenoid, a micron-sized, membraneless organelle of the chloroplast stroma (5, 6). Only one pyrenoid, from the green alga *Chlamydomonas reinhardtii*, has been studied in detail (7). Rubisco and the intrinsically disordered repeat protein essential pyrenoid component 1 (EPYC1) (8) demix in the chloroplast stroma via complex coacervation (9, 10) to form a liquid biomolecular condensate (11). The pyrenoid matrix is traversed by thylakoid tubules (12, 13) and contains on the order of 100 additional proteins at lower abundances (14, 15), some of which possess a defined Rubisco small subunit-binding motif (16, 17).

Microalgal Rubiscos do not share a common phylogeny but due to horizontal gene transfers belong to diverse green (Form IB), red (Form ID) (18), or even Form II (19) lineages. Their association with pyrenoids (20) indicates that the condensate has evolved convergently. Marine diatoms have been proposed to contribute 20% of global primary productivity (21), but molecular information on their “red-type” pyrenoids, which contain Form ID Rubisco, remains sparse (22, 23). The *Phaeodactylum tricornutum* pyrenoid presents as rod-shaped electron-dense moiety located in the center of the single elongated chloroplast (24–26). Two appressed thylakoids bisect the structure (20), and the lumen harbors a carbonic anhydrase that provides a CO_2_ point source (24, 27). The pyrenoid matrix not only contains Rubisco (20) but clusters of other enzymes including fructose bisphosphate aldolase (28) and multiple additional carbonic anhydrase isoforms (26).

We report the identification and characterization of a diatom Rubisco-binding protein pyrenoid component 1 (PYCO1), which contains prion-like domains (PLD). PYCO1 phase-separates homotypically and heterotypically with Rubisco. Repeating sticker motifs on PYCO1 are responsible for its intermolecular association and for binding Rubisco. The characterization of a convergently evolved pyrenoid will expand options available to synthetic biology efforts toward enhancing photosynthetic efficiencies by engineering the CO_2_-fixing reactions (29, 30).

## Results

### The Rubisco-Binding Protein PYCO1 Localizes to the Pyrenoid.

We raised a highly specific and sensitive peptide antibody against an epitope that is displayed on the surface of diatom Rubisco (*SI Appendix*, Fig. S1 *A* and *B*). Rubisco was isolated from soluble algal lysate by immunoprecipitation (*SI Appendix*, Fig. S1*B*) followed by analysis using tandem mass spectrometry (LC-MS/MS). A control experiment performed using beads not coupled to the antibody indicated very low background binding, but the corresponding sample was not analyzed by LC-MS/MS (*SI Appendix*, Fig. S1*B*). In three of five samples, the algae were exposed to dithiobis(succinimidyl propionate) prior to lysis in order to covalently stabilize interactions.

In these experiments, a total of 89 potential Rubisco-interacting proteins were identified (*SI Appendix,* Fig. S1*C* and Dataset S1). In all samples, Rubisco had the highest peptide coverage, and the pyrenoid-localized carbonic anhydrase PtCA1 (26) was always detected (Dataset S1). However, the dataset also contained high-scoring proteins unlikely to be associated with the pyrenoid, such as the cytosolic glyceraldehyde 3-phosphate dehydrogenase GapC2 (31), stressing the need for cautious validation.

**Fig. 1.**
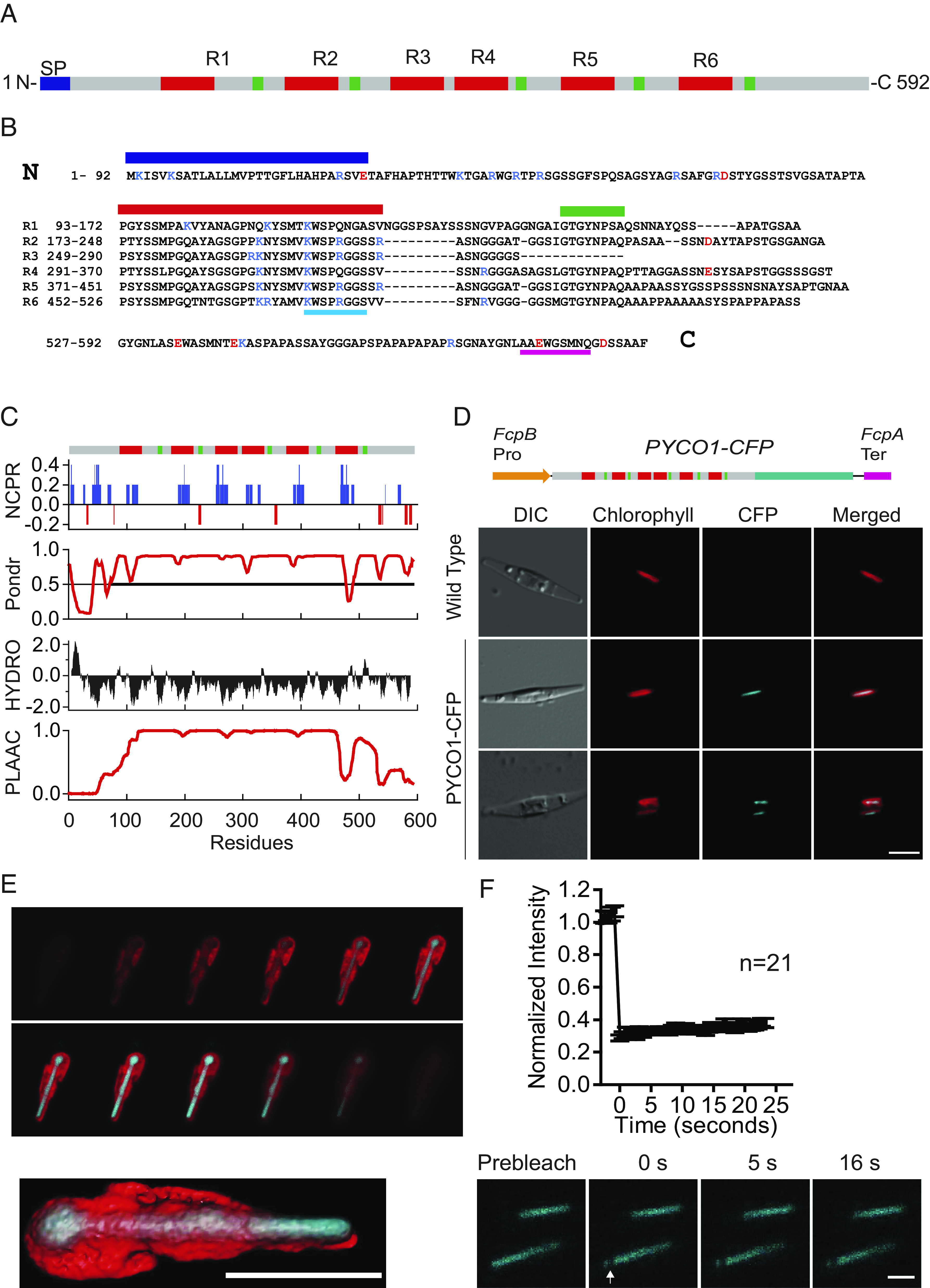
PYCO1 localizes to the diatom pyrenoid. (*A*) Schematic of the PYCO1 protein sequence. Predicted transit peptide and highly conserved repeat sequences are indicated in blue, red, and green, respectively. (*B*) Amino acid sequence of PYCO1. The predicted transit peptide is indicated using a blue bar. The six repeats are aligned, and the conserved sequences are indicated with green and red bars. Sequences shown to bind Rubisco in this study are underlined using cyan and magenta bars. Charged residues are indicated in blue (positive) and red (negative). (*C*) Bioinformatic analysis of the PYCO1 sequence reflecting net charge per residue (NCPR), disorder prediction (PONDR), hydropathy (HYDRO), and prion-like domains (PLAAC). (*D*) Schematic of PYCO1-CFP regulated by FcpB promoter (orange) and FcpA terminator (magenta). Microscope images of wild-type cells and PYCO1-CFP transformants. Differential interference contrast (DIC), chlorophyll autofluorescence, CFP fluorescence, and merged images are presented. (Scale bar, 10 μm.) (*E*) Selected confocal sections of PYCO1-CFP transformant and 3D reconstructed model of PYCO1 localization. (Scale bar, 5 μm.) (*F*) FRAP of PYCO1-CFP compartment, photobleaching was targeted at the tip, n = 21. Images reflect prebleach, 0, 5, and 16 s after photobleaching. (Scale bar, 2.5 μm.) Error bars indicate the SEM.

Rubisco condensation has recently been repeatedly linked to the process of liquid–liquid phase separation (LLPS) (32, 33), mediated by an intrinsically disordered repeat protein, where the repeats contain conserved motifs (8) or even folded domains (34) that function as multivalent Rubisco-binding sites or stickers (35–37). Phatr3_J49957 was detected in 4/5 samples and possessed features compatible with an LLPS scaffold protein. Amplification of the gene from genomic DNA revealed multiple amino acid substitutions and a 9-amino-acid insertion that were also found in expressed sequence tags (*SI Appendix,* Table S1). The protein contains six tandem repeats (R1-R6), each spanning ~80 amino acids except R3, which encodes the first half of a repeat (Fig. 1*A*). Similar to the green algal Rubisco linker protein EPYC1, Phatr3_J49957 was predicted to be mostly disordered, hydrophilic, and positively charged at physiological pH (pI = 10.03) (Fig. 1*C* and *SI Appendix,* Fig. S1*F*). Positively charged residues are clustered in the highly conserved N-terminal half of each repeat (Fig. 1 *A*–*C*). Unlike EPYC1, the majority of the sequence (residues 92 to 530) was classified as a PLD by the PLAAC algorithm (38) (Fig. 1*C* and *SI Appendix,* Fig. S1*F*), which is a common feature of condensate-forming proteins (39).

What follows implies that Phatr3_J49957’s functions similarly to EPYC1 in *P. tricornutum*. We propose the term PYCO1 (Pyrenoid Component 1) for Phatr3_J49957 as we do not demonstrate essentiality. BLAST searches to reveal homologs in other sequenced algal species were unsuccessful, with the only hit revealing a short PYCO1 isoform encoded by the same genome (Phatr3_J40791).

We used bacterial conjugation to transform *P. tricornutum* with the episome pPtPuc3*_FcpB_PYCO1ECFP_FcpA* encoding a fusion of PYCO1 and cyan fluorescent protein (PYCO1-ECFP) (40). The fluorescent signal localized to the center of the chloroplast stroma and presented as a rod ~6 μm long and <1 μm wide (Fig. 1*D* and *SI Appendix,* Fig. S1*D*). Reconstructed 3D models further demonstrated that the PYCO1-ECFP signal was embedded within the chlorophyll autofluorescence. This localization pattern is consistent with pyrenoid localization and dimensions determined by electron microscopy (24, 25, 41) (Fig. 1*E* and *SI Appendix,* Fig. S1 *D* and *E*). PYCO1-ECFP did not recover within 25 s when a 0.25-μm^2^ region was photobleached. This indicates that the diatom pyrenoid is less dynamic compared to the liquid-like *Chlamydomonas* pyrenoid where near-complete recovery of EPYC1-Venus was observed within this time frame (11) (Fig. 1*F* and Movie S1).

### PYCO1 Undergoes Homotypic LLPS In Vitro.

To understand the Rubisco–PYCO1 interaction, we optimized the recombinant production and purification of PYCO1 protein [residues 31 to 592, lacking the chloroplast transit peptide as predicted by ASAFind (42)] and its C-terminal mEGFP fusion protein, PYCO1-GFP, in *Escherichia coli* (*SI Appendix,* Fig. S2*A*). Extensive problems with proteolysis were eventually overcome. In the final protocol, the resuspended *E. coli* biomass was boiled prior to lysis, and proteins were purified from inclusion bodies that were redissolved in 8 M urea (*SI Appendix,* Fig. S2*B*). The inclusion of a C-terminal His_6_ tag permitted affinity chromatography, and an N-terminal FLAG epitope was used to track the full-length protein during purification.

**Fig. 2.**
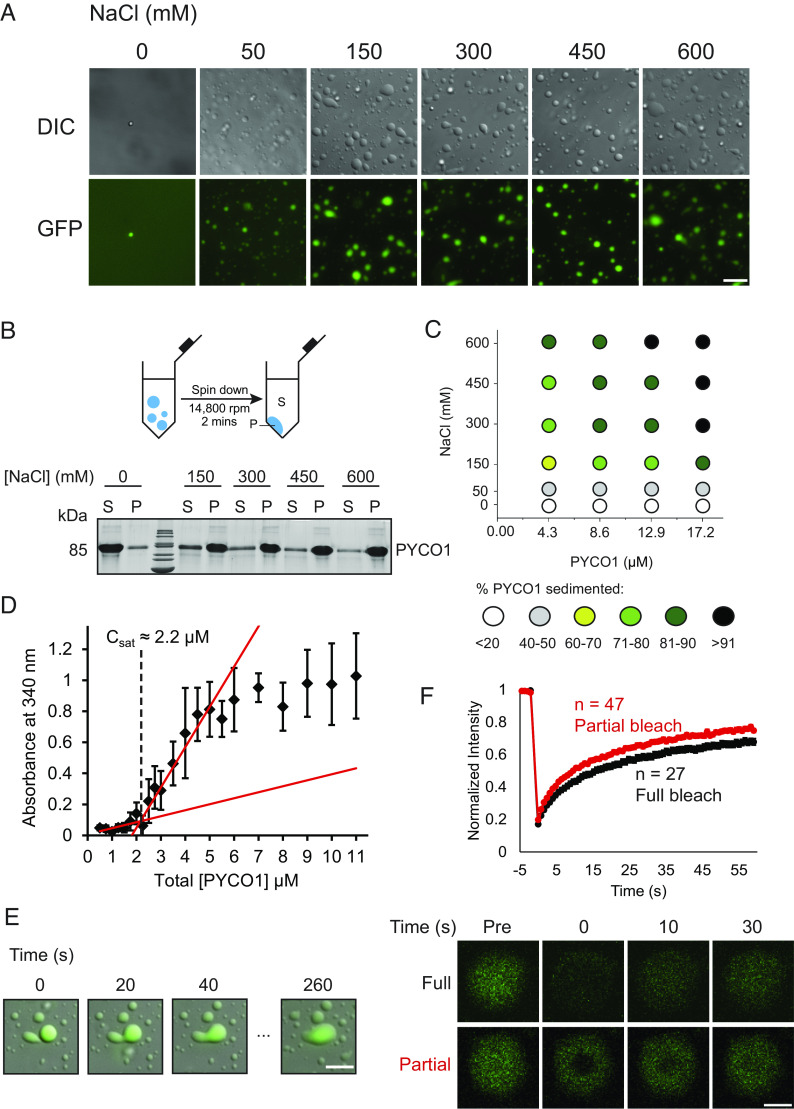
Homotypic phase separation of PYCO1. (*A*) Representative microscopic images of PYCO1 condensates in DIC and GFP channels. 8.6 µM PYCO1 (with 5% PYCO1-GFP) in 20 mM Tris pH 8.0. (Scale bar, 15 µm.) (*B*) Schematic of sedimentation experiments showing 8.6 µM PYCO1 accumulating in pellet fraction in response to NaCl concentrations. (*C*) Phase diagram of PYCO1 based on sedimentation experiments, showing phase behavior of PYCO1 as a function of salt and protein concentration. (*D*) Turbidity measurements of PYCO1 at 340 nm. C_sat_ of PYCO1 was calculated to be approximately 2.2 µM. Error bars represent SD. (*E*) Coalescence event of PYCO1 condensates. (Scale bar, 10 µm.) (*F*) Bleaching experiments on PYCO1 using a laser scanning confocal microscope. Condensates of approximately 3 µm in diameter were selected for full bleach experiments, while bleaching diameter was set to 1 µm in partial bleach experiments. 8.6 µM PYCO1 (with 5% PYCO1-GFP) in 20 mM Tris pH 8.0 and 150 mM NaCl. (Scale bar, 2 µm.) Error bars represent SEM.

Prior to analysis, PYCO1 was dialyzed into 20 mM Tris-HCl pH 8.0. PYCO1 was mixed with 5% PYCO1-GFP, and incubation in the presence of at least 50 mM NaCl resulted in the immediate formation of spherical, fluorescent condensates (Fig. 2*A* and *SI Appendix,* Fig. S2 *C* and *D*). PYCO1 droplets could be sedimented by centrifugation (Fig. 2*B*). The assay was used to build a phase diagram, which revealed that demixing was favored at increased protein and salt concentrations (Fig. 2*C* and *SI Appendix,* Fig. S2*E*). The condensates could be repartitioned by supernatant exchange or dissolved by dilution of the salt (*SI Appendix,* Fig. S2 *F* and *G*).

These findings suggest that the PYCO1–PYCO1 protein–protein interactions (PPIs) driving the phase separation are not disrupted by increased ionic strength. Charge screening may overcome the repulsion between the cationic PYCO1 molecules permitting intermolecular sticker interactions. Turbidity measurements revealed that the saturation concentration (C_sat_) of PYCO1 at 150 mM NaCl was approximately 2 µM (Fig. 2*D*). Using quantitative confocal microscopy, the concentration of the light phase (C_light_) and dense phase (C_dense_) was determined to be approximately 5.7 µM and 575 µM, respectively, corresponding to a partition coefficient (PC) of ~100 at 150 mM NaCl (*SI Appendix,* Fig. S3 *A–C* and Table S2). PYCO1 droplets exhibited their liquid-like nature through coalescence events (Fig. 2*E* and Movie S2) and also by FRAP experiments with a recovery half-time of 9 ± 0.8 s on fully bleached droplets of 3 µm diameter (Fig. 2*F*).

Hence, PYCO1, like other proteins containing PLDs, was able to phase separate in vitro potentially enabling a role as a scaffold protein that can recruit additional components of the pyrenoid.

### Diatom Rubisco Specifically Partitions into PYCO1 Condensates.

**Fig. 3.**
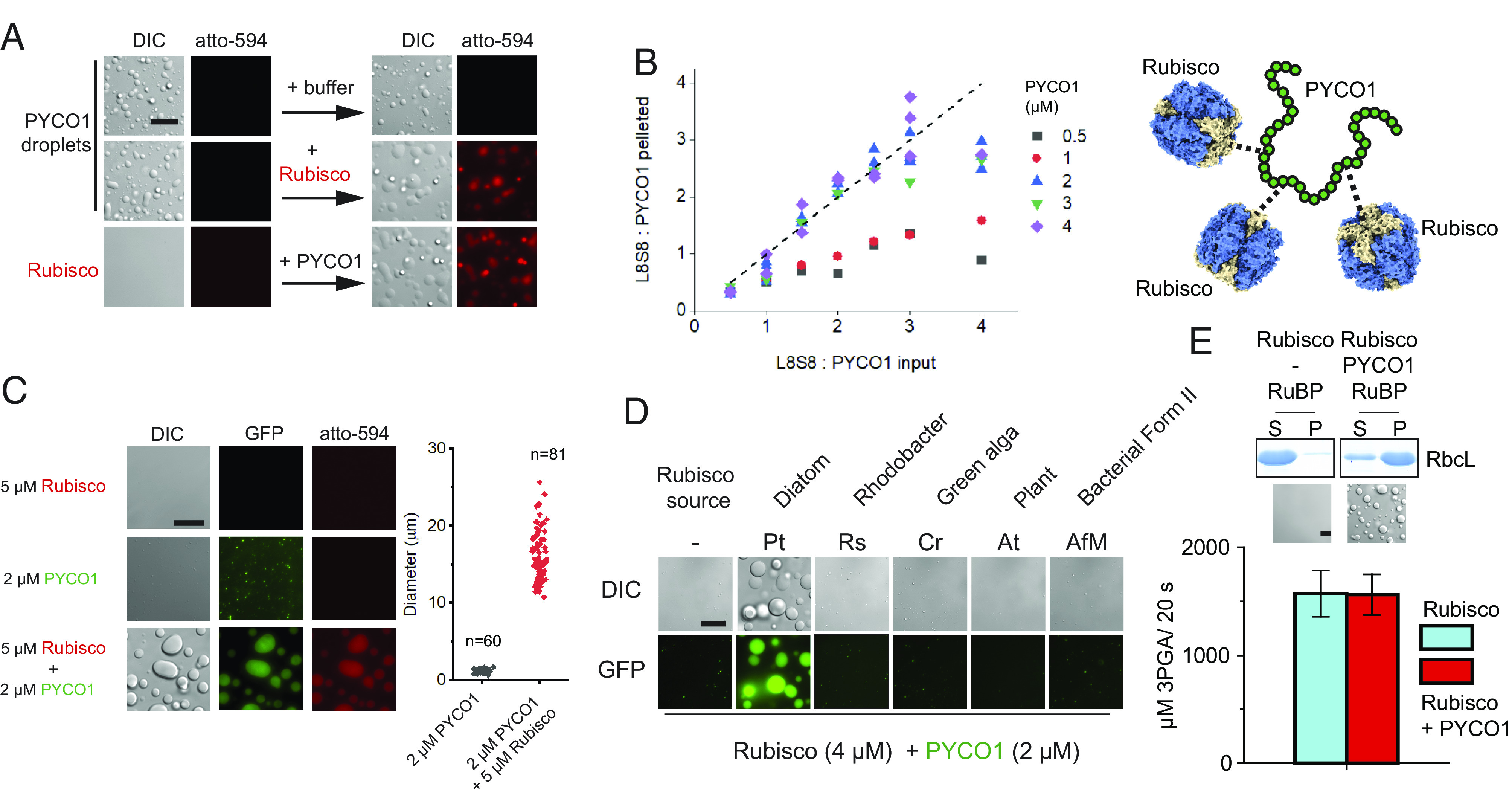
Diatom Rubisco specifically partitions into PYCO1 condensates. (*A*) Preformed PYCO1 droplets recruit Rubisco. 8.6 µM PYCO1 and 1.1 µM Rubisco (0.3% atto-594-labeled). (*B*) Sedimentation analysis of PYCO1–Rubisco condensates. Saturation is reached at three Rubiscos per PYCO1 molecule. (*C*) Condensates saturated with Rubisco increase in size. Rubisco (0.6% atto labeled) and PYCO1 (5% PYCO1-GFP). (*D*) PYCO1 can only form droplets with diatom Rubisco. Rs, *Rhodobacter sphaeroides*; Cr, *Chlamydomonas reinhardtii*; At, *Arabidopsis thaliana*; AfM, *Acidithiobacillus ferrooxidans* Form II Rubisco. (*E*) Rubisco activity in the condensates is unaffected. (Scale bars, 15 µm.)

Could PYCO1 condensates function as a Rubisco-binding scaffold? At 150 mM NaCl, preformed PYCO1 droplets efficiently recruited fluorescent PtRubisco (purified algal Rubisco (*SI Appendix,* Fig. S4*A*) spiked using 0.3% atto-594-labeled PtRubisco) irrespective of the order of addition (Fig. 3*A*). Titration followed by sedimentation assays suggested that per molecule of PYCO1, three Rubisco (L_8_S_8_) complexes could be partitioned into the condensate (Fig. 3*B* and *SI Appendix,* Fig. S4*B*). When PYCO1 concentrations below 2 µM were employed, Rubisco sedimentation was inefficient (Fig. 3*B* and *SI Appendix,* Fig. S4*B*).

Rubisco partitioning was salt sensitive, and at 600 mM NaCl, the enzyme was mostly found in the light phase. This indicated that in contrast to PYCO1–PYCO1 interactions, the PPIs between Rubisco and PYCO1 include electrostatic contributions (*SI Appendix,* Fig. S4*C*).

Heterotypic condensates formed at high Rubisco to PYCO1 ratios were much larger than the respective homotypic PYCO1 condensates (Fig. 3*C* and *SI Appendix,* Fig. *S*4*D*), consistent with the increased mass of the heterotypic dense phase. Multiple eukaryotic and prokaryotic Rubiscos were assayed for their ability to form a heterotypic condensate with PYCO1, but only the *P. tricornutum* enzyme was competent, indicating highly specific PPIs (Fig. 3*D* and *SI Appendix,* Fig. *S*5*A*). Homotypic PYCO1 condensates also did not partition heterologous Rubiscos, with the exception of the bacterial red-type enzyme from *Rhodobacter sphaeroides* (*SI Appendix,* Fig. *S*5*A**)*. Radiometric ^14^CO_2_ fixation assays indicated that condensed diatom Rubisco was fully functional (Fig. 3*E*). Condensate appearance or Rubisco partitioning (*SI Appendix,* Fig. *S*5 *B* and *C*) was not affected by activated Rubisco or the inclusion of Rubisco’s substrates (4 mM ribulose 1,5-bisphosphate, and 20 mM NaHCO_3_).

### Rubisco Reduces the Mobility of Condensate Components.

**Fig. 4.**
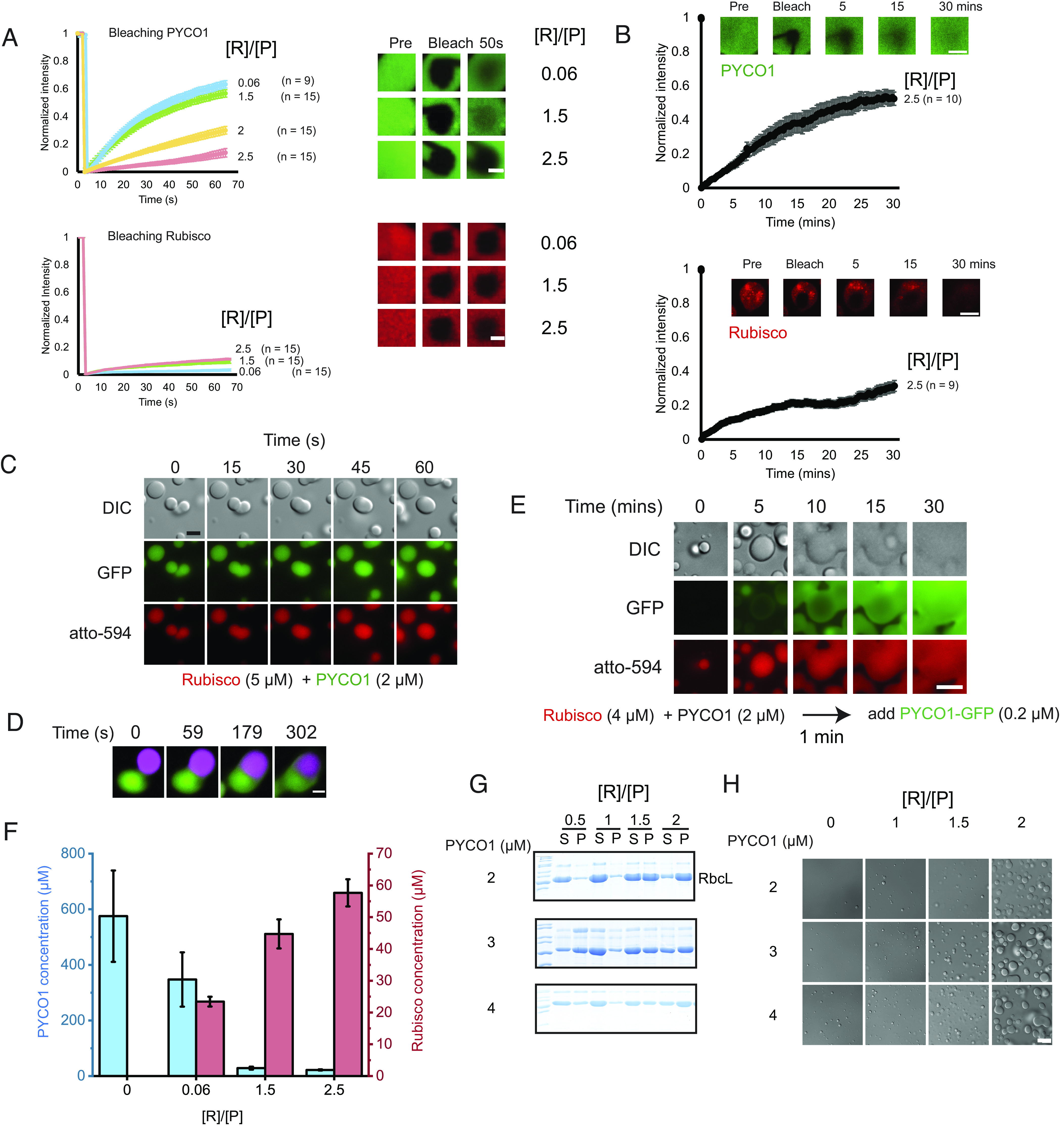
Condensate composition determines its properties. (*A* and *B*) FRAP experiments of heterotypic PYCO1–Rubisco condensates. [R]/[P] = [Rubisco]/[PYCO1]. PYCO1 mobility decreases as the proportion of Rubisco is increased. Rubisco always has low mobility. PYCO1 concentration used was 2 µM, except when [R]/[P] = 0.06 (0.54 µM Rubisco/ 8.6 µM PYCO1). (Scale bars, 1 µm *A* and 10 µm *B*.) Error bars indicate the SEM. (*C*) Coalescence of heterotypic PYCO1–Rubisco condensates. [R]/[P] = 2.5. (Scale bar, 15 µm.) (*D*) PYCO1–mRuby and PYCO1–GFP heterotypic condensates ([R]/[P] = 2.5) were prepared separately and mixed. PYCO1 mixes very slowly upon coalescence. (Scale bar, 5 µm.) (*E*) PYCO–GFP can invade preformed PYCO1–Rubisco droplets ([R]/[P] = 2) on a minute timescale. (Scale bar, 10 µm.) (*F*) The concentration of PYCO1 in the droplets drastically decreases with increasing Rubisco:PYCO1 ratios. PYCO1 concentration was measured using fluorescence microscopy, and Rubisco concentration was derived by densitometric quantification of a pelleted Rubisco large subunit. Error bars indicate the SEM. (*G* and *H*) Sedimentation analysis (*G*) and microscopy (*H*) show that condensate formation is suppressed when [Rubisco] and [PYCO1] are equimolar. (Scale bar, 15 µm.)

We performed FRAP experiments on heterotypic condensates loaded with various Rubisco stoichiometries. Strikingly, PYCO1 mobility decreased sharply once the Rubisco content increased beyond 1.5 Rubiscos per PYCO1 (Fig. 4*A*). At near-saturating stoichiometries of 2.5 Rubiscos to 1 PYCO1 molecule, recovery of the scaffold protein became very slow with a recovery half-time of ~ 9 min (Fig. 4 *A* and *B* and *SI Appendix,* Fig. S5*D*). Rubisco recovery was slow in all conditions sampled. This finding can be explained by Rubisco molecules contributing to a network formed by high-affinity Rubisco–PYCO1 interactions, whereas excess unbound PYCO1 molecules are able to diffuse more freely within the condensate.

Consistent with slow diffusive mixing of Rubisco-bound PYCO1, both droplet fusion and relaxation could be observed on a minute timescale (Fig. 4*C* and *SI Appendix,* Fig. *S5E*). When heterotypic droplets labeled with two different fluorescent PYCO1 fusion proteins were mixed, the signals remained in distinct sectors for 5 min following droplet fusion (Fig. 4*D*, *SI Appendix,* Fig. S5*F*, and Movie S3). When PYCO1–Rubisco condensates were preformed, and the PYCO1-GFP label was added subsequently, slow diffusion into the condensates was observed (Fig. 4*E*). Collectively, these observations indicate that there are high-affinity interactions between PYCO1 and Rubisco, but the network can still rearrange and is not arrested.

Using fluorescence microscopy, we measured the PYCO1 concentrations in droplets formed under variable stoichiometries. PYCO1 concentration was ~600 µM in homotypic droplets, and this value dropped dramatically to ~30 µM when the ratio of Rubisco to PYCO1 was 2.5 (*SI Appendix,* Table S2). Combining these results with the densitometric analysis (Fig. 3*B* and *SI Appendix,* Fig. *S4B*) permitted the estimation of Rubisco concentrations (*SI Appendix,* Table S3). The heterotypic droplets contained ~60 µM/ ~30 mg/mL of Rubisco (Fig. 4*F*). Formation of heterotypic condensates resulted in lower light phase concentrations for PYCO1 (*SI Appendix,* Table S2). The variability in saturation concentration and partition coefficients is a feature of heterotypic condensates that emerges as a result of the different valencies and affinities of the interactions involved (43, 44).

Rubisco:PYCO1 stoichiometry could strongly affect phase separation. At equimolar concentrations, both proteins were mostly found in the soluble fraction, and droplets were small (Fig. 4 *G* and *H* and *SI Appendix, Fig. S4B*). As the network dominated by PYCO1–PYCO1 interaction transitions to a PYCO1-Rubisco network, the 1:1 stoichiometry results in a dramatic change in the phase diagram. Only when more Rubisco-binding sites are added to the system, the condensates become stabilized.

### Identification of the Small Subunit-Binding PYCO1 Stickers.

**Fig. 5.**
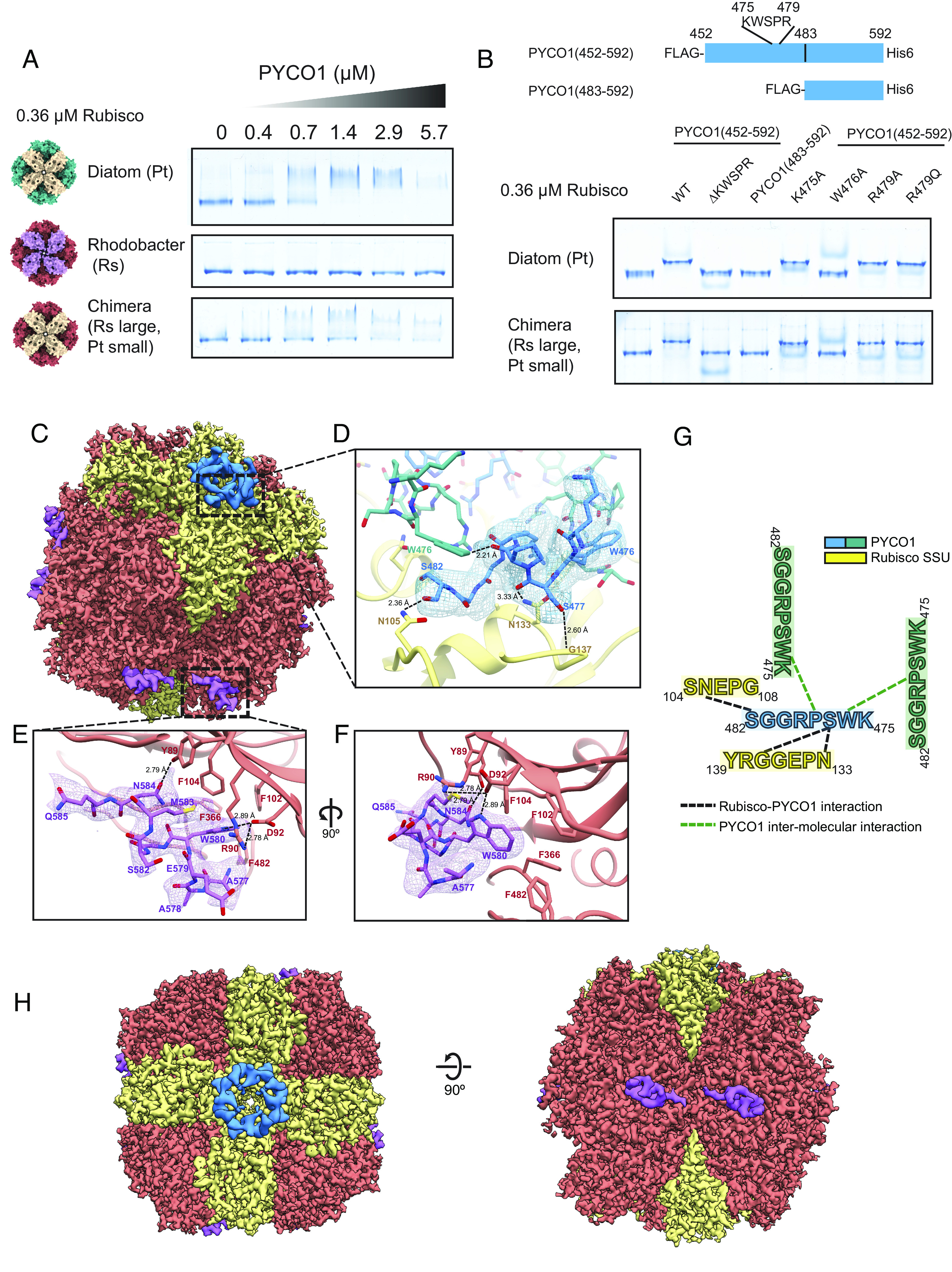
PYCO1 repeat motifs oligomerize and bind to the Rubisco small subunit. (*A*) PYCO1 interacts with the diatom small subunit. The native-PAGE gel shift assay indicates that PYCO1 interacts with Rubisco containing the diatom small subunit (Pt small). (*B*) A single repeat fragment of PYCO1 (residues 452 to 592) can interact with Rubisco. Mutation of W476 in the repeating KWSP motif abolishes the interaction. (*C*) Density map of PtRubisco bound to PYCO1. Large subunits are colored in salmon, and small subunits are colored in khaki. PYCO1 binds at two distinctive positions on PtRubisco. On the large subunit, PYCO1 is colored in magenta. On the small subunit, PYCO1 is colored in blue. (*D*) Interactions between PYCO1 fragment at the small subunit interface. PYCO1 is colored in blue, and adjacent PYCO1 molecules are colored in teal. PYCO1 interaction with SSU is held together by hydrogen bond networks involving S477 and S482 on PYCO1 with N105, N133, and G137 on PtRubisco SSU. Intermolecular PYCO1 interactions between W476 and P478 stabilize the PYCO1 complexes. (*E* and *F*) Interactions between PYCO1 fragments at the large subunit interface. PYCO1 is colored in magenta. Hydrogen bonds involving N584 and W580 on PYCO1 with Y89 and D92 on PtRubisco LSU. Interactions are further stabilized by hydrophobic interactions between W580 on PYCO1 with F102, F104, F366, and F482 on the LSU. (*G*) Cartoon representation of the interactions at the SSU. (*H*) Top and side views of the density map.

The addition of recombinant PYCO1 protein brought about a concentration-dependent native gel shift of diatom but not of proteobacterial *Rhodobacter sphaeroides* Rubisco (Fig. 5*A*). However, when we used a chimeric Rubisco composed of proteobacterial large, and diatom small subunits (RsLPtS) (*SI Appendix,* Fig. *S6A*), the gel shift was restored, indicating that the responsible PYCO1-binding site localizes to the Rubisco small subunit. Interestingly, although RsLPtS–PYCO1 condensates could be sedimented, no regular spherical droplets were observed. Instead, the condensates formed aggregated clusters of small droplets that failed to fuse (*SI Appendix,* Fig. S6 *B–D*).

To localize the Rubisco small subunit-binding site of PYCO1, we produced a series of fragments (*SI Appendix,* Fig. *S6A*) and used the gel shift assay to assess Rubisco binding. PYCO1(452 to 592), which encompassed the final repeat and C-terminus, reduced the electrophoretic mobility of diatom Rubisco, indicating the formation of a defined complex (Fig. 5*B*). In contrast, variants of the fragment where the repeating KWSPR motif was deleted, or W476 substituted with alanine, did not alter diatom Rubisco migration in this assay (Fig. 5*B*). This was also the case for the fragment PYCO1(483 to 592), which encoded the C-terminal 109 residues that follow the last repeat (Fig. 5*B*).

These data suggested that PYCO1 binds to the Rubisco small subunit via a small linear interacting motif (45) that includes a critical tryptophan residue.

### The Structure of a Rubisco–PYCO1 Fragment Complex Reveals a Sticker Tetrad.

We determined the structure of the Rubisco–PYCO1(452 to 592) complex using cryo–electron microscopy (Fig. 5 *C–H* and *SI Appendix,* Fig. *S*7). The resulting density map revealed that distinct PYCO1 motifs bind to both the small (Fig. 5*D* in blue; 2.6 Å resolution) and the large subunits (Fig. 5 *E* and *F* in magenta; 2.5 Å resolution) of Rubisco (2.0 Å resolution).

The *P. tricornutum* Rubisco structure overall was highly similar to the diatom Rubisco structures reported recently (46), with rmsd of 0.865 Å for all Cα atoms of the large subunit compared to the *Thalassiosira antarctica* Rubisco structure (PDB:5MZ2). Density for several posttranslational modifications could also be identified, all of which were described in the earlier diatom Rubisco structures (*SI Appendix,* Fig. S8).

Four instances of the previously identified sticker motif “KWSPRGGS” could be modeled into the density map surrounding Rubisco’s central solvent channel formed by the small subunits (Fig. 5*D*). The motifs adopt a helical conformation and collectively form a square-shaped plug of the central solvent channel (Fig. 5 *C* and *H*). The indole group of each W476 binds to a groove formed by the “GGS” of the adjacent sticker, connecting the corners of the square. Because mutation of W476 abolished Rubisco binding (Fig. 5*B*), it appears likely that the interaction between at least two PYCO1 stickers is essential for heterotypic condensate formation.

The interaction between the small subunits of Rubisco and the PYCO1 sticker tetrad is mediated by a network of hydrogen bonds. The side chain of SSU N105, which is located in the loop connecting βD and βE, hydrogen bonds with the side chain of PYCO1 S482. Two more residues in the SSU C-terminal extension form hydrogen bonds to the PYCO1 motif. The N133 carboxamide forms an interaction with the backbone of PYCO1 S477 (Fig. 5 *D* and *G*). PYCO1 S477 has a second interaction with the G137 backbone via its side chain.

The structure revealed an additional interaction of the Rubisco large subunit with the PYCO1 fragment, the motif “AAEWGSMNQ” found near the C-terminus of PYCO1 (residues 577 to 585). The element adopted a helical conformation and bound to a largely hydrophobic cleft formed between the two domains of the large subunit near Rubisco’s dimer–dimer interface (Fig. 5 *C*, *E*, *F*, and *H*). There is a hydrogen bond between the side chains of PYCO1 N584 and LSU Y89. The indole group of PYCO1 W580 is inserted into a hydrophobic pocket formed by LSU residues F102, F104, L361, F366, and F482. W580 also participates in a hydrogen bond to D92 (Fig. 5 *E* and *F*). The C-terminus of PYCO1 harbors an additional similar motif (ASEWASMNT residues 532 to 540) that may also bind in this way. However, the interaction between Rubisco and these motifs is relatively weak because mutating the SSU-binding motif of PYCO1(452 to 592) was sufficient to eliminate the observed native PAGE shift. The C-terminal fragment containing these motifs did not alter Rubisco’s electrophoretic mobility (Fig. 5*B*).

### A Tyrosine-Repeat Motif Is Essential for PYCO1 Condensate Formation.

**Fig. 6.**
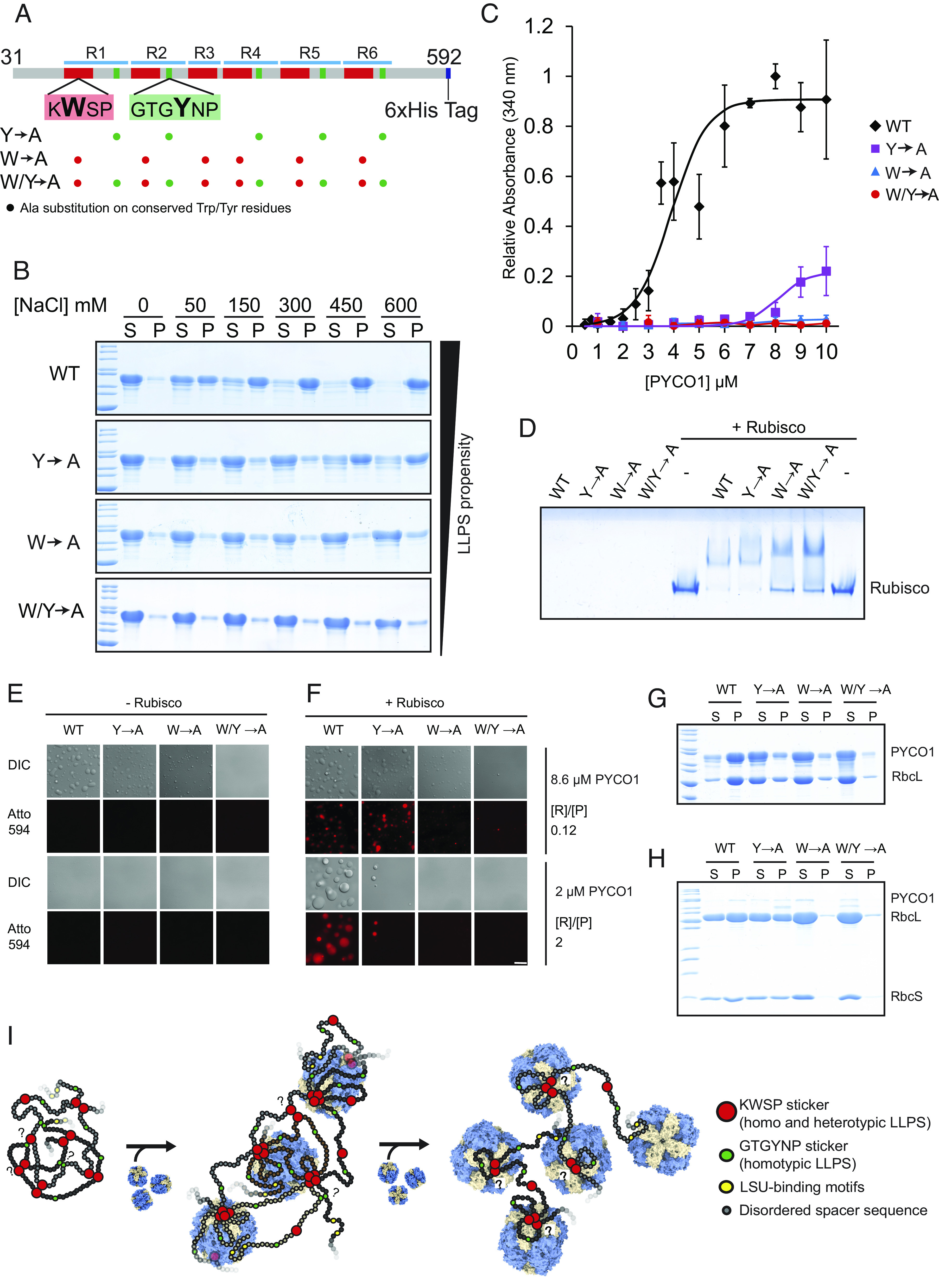
PYCO1 condensate formation involves aromatic sticker residues found in repeating motifs. (*A*) Scheme showing the two sticker motifs and summarizing the PYCO1 variants used. (*B*) Sedimentation analysis of PYCO1 variants (8.6 µM) in 20 mM Tris–HCl pH 8.0 using the indicated NaCl concentrations. (*C*) Turbidity measurements of PYCO1 variants at varying concentrations were carried out at 20 mM Tris–HCl, pH 8.0, and 150 mM NaCl. Error bars indicate SD. (*D*) Native-PAGE gel shift assays containing 0.36 µM Rubisco and 2.9 µM PYCO1 variants. (*E* and *F*) Homotypic (*E*) and heterotypic (*F*) condensates of the PYCO1 variants were formed in 20 mM Tris–HCl, pH 8.0, and 150 mM NaCl. (Scale bar, 15 µm.) (*G* and *H*) Sedimentation analysis of the heterotypic condensates formed at low (0.12, *G*) and high (2, *H*) Rubisco:PYCO1 ([R]/[P]) ratios. (*I*) PYCO1 forms a dense phase mediated by interspecific and intraspecific protein–protein interactions that involve aromatic residues in the repeating KWSP and GTGYNP motifs. It is possible that KWSP sticker oligomers exist. Rubisco molecules can enter the PYCO1 condensate and are immobilized by high-affinity interactions involving KWSP sticker tetrads on the apex and bottom of the holoenzyme. When Rubisco is saturating, both PYCO1 and Rubisco are involved in a system-spanning network resulting in low component mobility.

The observed homotypic phase separation of PYCO1 predicts the existence of multivalent stickers that mediate homotypic interactions (36). Inspection of the low-complexity region of PYCO1 revealed the presence of five completely conserved GTGYNP motifs found in all repeats except for the truncated R3 (Fig. 1*A*). Given the emerging importance of tyrosine residues in mediating interactions leading to LLPS in multiple systems (47–50), we mutated the corresponding five tyrosine residues to alanine and produced PYCO1(Y→A). We also produced PYCO1(W→A), where all six tryptophan residues of the SSU-binding KWSPR motif were substituted with alanine, and PYCO1(W/Y→A), where all eleven mentioned aromatic residues were substituted with alanine (Fig. 6*A*).

When compared to the wild-type protein, all three variants were strongly impaired in their ability to form homotypic condensates (Fig. 6 *B*, *C*, and *E*). PYCO1(Y→A) phase separation could still be observed at high salt or protein concentrations (Fig. 6 *B* and *C*). The strong impairment of PYCO1(W→A) homotypic phase separation revealed that the Rubisco-binding motif is also involved in homotypic phase separation and raises the possibility that the sticker–sticker interaction observed in the Rubisco–PYCO1(452 to 592) structure already forms in the homotypic condensate.

Next, we assessed heterotypic condensate formation. The interaction of the Y→A variant with Rubisco was comparable to wild type, as assessed by the native-PAGE gel shift assay (Fig. 6*D*). As expected from our structure, this was not true for W→A and W/Y→A, which did not completely shift Rubisco in this assay. Heterotypic condensate formation and Rubisco recruitment were robust for Y→A but essentially eliminated for W→A and W/Y→A (Fig. 6 *F*–*H*).

In summary, our data indicate that both the tyrosine residues embedded in the repeating GTGYNP motifs and the KWSPR/Q tryptophans are involved in homotypic condensate formation and thus will be involved in intermolecular and intramolecular PYCO1 interactions. Formation of the heterotypic condensate requires the tryptophan of the SSU-binding motif to be intact. The behavior of the Y→A variant suggests that homotypic phase separation is not essential for heterotypic condensate formation.

## Discussion

We present a biochemical framework for formation of the Form ID Rubisco condensates found in diatoms, representatives of the red plastid lineage. The identification and characterization of PYCO1 indicates that the convergently evolved red pyrenoid matrix, like its counterpart in green algae (8, 11), is formed by LLPS mediated by an intrinsically disordered repeat protein that contains multiple sticker motifs that bind to the Rubisco large and small subunits.

The green algal EPYC1 protein does not form homotypic condensates under comparable conditions but requires the presence of interacting Rubisco to demix (9). In contrast, PYCO1 is a protein containing PLDs that can undergo homotypic phase separation. The underlying interactions involve aromatic residues, and the homotypic condensates can subsequently specifically bind diatom Rubisco. The green algal Rubisco linker protein EPYC1 possesses five evenly spaced helical sticker motifs, which specifically bind to the two α-helices of the small subunit, where one sticker will bind one small subunit. The EPYC1 sticker forms salt bridges to both helices via weak interactions (10). The resulting droplets are spherical, and the pyrenoid matrix components are highly mobile (9, 11). In PYCO1, the six small subunit-binding stickers are unevenly distributed (Fig. 1*A*). Four stickers can interact to form a tetrad and stably bind to the small subunits lining Rubisco’s central solvent channel via a hydrogen bond network. The interaction between the stickers is mediated by a critical tryptophan residue. Sticker oligomerization stabilizes the interaction between PYCO1 and Rubisco resulting in high-affinity interactions and a less dynamic Rubisco condensate.

Variations of the EPYC1 sticker motif are found in other *Chlamydomonas* pyrenoid components, possibly providing a universal pyrenoid sticker that appears to organize the green pyrenoid (16). In contrast, we did not encounter the KWSPR/Q sticker motif in other *Phaeodactylum* proteins, other than in the short PYCO1 isoform Phatr3_J40791. Variations of the encountered large subunit-binding helix are found in other proteins and may fulfill this role instead.

Formation of the dense (~30 mg/mL) homotypic PYCO1 condensate was driven by salt and could be disrupted by mutation of aromatic residues, including the tryptophan critical for Rubisco binding. PYCO1 is mobile in the homotypic condensate, indicating that the underlying interactions are weak (Fig. 6*I*). It is possible that the sticker oligomerization observed in the Rubisco-bound state already exists and stabilizes the homotypic condensates. In addition, we note a similarity to the behavior of mussel foot proteins (mfps). These are positively charged proteins containing aromatic residues that form homotypic condensates with increasing salt. The salt reduces the electrostatic repulsion between mfps but does not disrupt the π–cation interactions that drive the assembly (51, 52).

As Rubisco is added to the condensate, its properties change. The PYCO1 concentration in the dense phase decreases dramatically, indicating that PYCO1–PYCO1 interactions are being substituted for PYCO1–Rubisco interactions (Fig. 6*I*). At a 1:1 stoichiometry, the condensate is destabilized. The sticker stoichiometry observed in the structure suggests that four PYCO1 molecules (with 24 KWSP motifs) would be able to completely saturate the small subunit-binding sites of three Rubisco particles. This ratio may thus favor the formation of stable smaller oligomers. This concept has been theoretically explored as a “magic-number effect” in the context of biomolecular condensates previously (11, 53). At high Rubisco/PYCO1 ratios, PYCO1 mobility is drastically reduced, indicating that all molecules are incorporated into the heterotypic network. In the heterotypic condensate corresponding to 2.5 Rubisco molecules (8 × 2.5 = 20 small and 20 large subunit-binding sites) to 1 PYCO1 (six small subunit stickers and two large subunit stickers), only a fraction of Rubisco-binding sites would be occupied. As PYCO1 small subunit sticker oligomerization appears critical, only one side of the Rubisco particle may thus be bound in the observed network (Fig. 6*I*).

The *Phaeodactylum* pyrenoid is not spherical but adopts a rod-like shape, and PYCO1-ECFP has low mobility (Fig. 1 *E* and *F*). We hypothesize that the viscous material properties of the Rubisco-saturated PYCO1 condensate resemble the algal pyrenoid matrix. This may permit the compartment, which is organized around a central thylakoid membrane (24), to maintain its distinctive rod-shaped structure. At the same time, changing the stoichiometry of matrix components provides one ready mechanism to change the properties of the compartment (54), and this may then facilitate processes such as pyrenoid division (11).

## Materials and Methods

Detailed methods and protocols on plasmid construction, protein purification, algal biology, immunoprecipitation and mass spectrometry, light microscopy, biochemical assays, and cryo–electron microscopy are provided in *SI Appendix*. Most proteins were produced recombinantly in *E. coli*, except for Rubisco, which was purified directly from diatoms. *P. tricornutum* was transformed by bacterial conjugation (40).

## Supplementary Material

Appendix 01 (PDF)

Dataset S01 (XLSX)

Dataset S02 (XLSX)

Movie S1. **FRAP of diatom cells expressing PYCO1-CFP**. Photobleaching of a *P. tricornutum* cell carrying pPtPuc3*FcpBPYCO1ECFPFcpA*. Fluorescently tagged PYCO1 was imaged and bleached at 458 nm and 405 nm. Bleached cells were allowed to recover for 25 seconds. ECFP channel is shown first, followed by the brightfield channel.

Movie S2. **Coalescence of PYCO1 homotypic condensates**. 8.6 μM of PYCO1 (5% PYCO1-mEGFP) was prepared in 20 mM Tris-HCl pH 8.0 and 150 mM NaCl in 5 μL volume. Condensates were imaged using DIC and GFP channels and merged.

Movie S3. **Coalescence of PYCO1-Rubisco heterotypic condensates**. 2 μM of PYCO1 (5% PYCO1-mEGFP, or 5% PYCO1-mRuby) and 5 μM of Rubisco was prepared in 20 mM Tris-HCl pH 8.0 and 150 mM NaCl in 5 μL volume. Condensates were imaged using GFP and mCherry channels and subsequently merged. Two video sets are shown consecutively.

## Data Availability

Data acquired and analysed during this study are included in this manuscript or available at https://researchdata.ntu.edu.sg/dataverse/cajar (55). Density maps from cryo-electron single particle analysis have been deposited into the Electron Microscopy Data Bank with the accession code EMDB-33887 (56) EMDB-35166 (57) EMDB-35159 (58) and EMDB-35158 (59) Molecular models have been deposited into the Protein Data Bank under the accession code PDB 7YK5 (60). The mass spectrometry proteomics data have been deposited to the ProteomeXchange Consortium via the PRIDE partner repository (61) with the dataset identifier PXD027027 (62).

## References

[1] C. B. Field, M. J. Behrenfeld, J. T. Randerson, P. Falkowski, Primary production of the biosphere: Integrating terrestrial and oceanic components. Science **281**, 237–240 (1998). 9657713 10.1126/science.281.5374.237

[2] M. R. Badger , The diversity and coevolution of Rubisco, plastids, pyrenoids, and chloroplast-based CO_2_-concentrating mechanisms in algae. Canadian J. Botany-Revue Canadienne de Botanique **76**, 1052–1071 (1998).

[3] J. R. Reinfelder, Carbon concentrating mechanisms in eukaryotic marine phytoplankton. Ann. Rev. Mar Sci. **3**, 291–315 (2011). 10.1146/annurev-marine-120709-142720 21329207

[4] M. Meyer, H. Griffiths, Origins and diversity of eukaryotic CO2-concentrating mechanisms: lessons for the future. J. Exp. Botany **64**, 769–786 (2013). 23345319 10.1093/jxb/ers390

[5] M. T. Meyer, C. Whittaker, H. Griffiths, The algal pyrenoid: Key unanswered questions. J. Exp. Botany **68**, 3739–3749 (2017). 28911054 10.1093/jxb/erx178

[6] J. Barrett, P. Girr, L. C. M. Mackinder, Pyrenoids: CO2-fixing phase separated liquid organelles. Biochim. Biophys. Acta Mol. Cell Res. **1868**, 118949 (2021). 33421532 10.1016/j.bbamcr.2021.118949

[7] T. Wunder, Z. G. Oh, O. Mueller-Cajar, CO2 -fixing liquid droplets: Towards a dissection of the microalgal pyrenoid. Traffic **20**, 380–389 (2019). 31001862 10.1111/tra.12650

[8] L. C. M. Mackinder , A repeat protein links Rubisco to form the eukaryotic carbon-concentrating organelle. Proc. Natl. Acad. Sci. U.S.A. **113**, 5958–5963 (2016). 27166422 10.1073/pnas.1522866113 PMC4889370

[9] T. Wunder, S. L. H. Cheng, S. K. Lai, H. Y. Li, O. Mueller-Cajar, The phase separation underlying the pyrenoid-based microalgal Rubisco supercharger. Nat. Commun. **9**, 5076 (2018). 30498228 10.1038/s41467-018-07624-w PMC6265248

[10] S. He , The structural basis of Rubisco phase separation in the pyrenoid. Nat. Plants **6**, 1480–1490 (2020). 33230314 10.1038/s41477-020-00811-y PMC7736253

[11] E. S. Freeman Rosenzweig , The eukaryotic CO2-concentrating organelle is liquid-like and exhibits dynamic reorganization. Cell **171**, 148–162.e119 (2017). 28938114 10.1016/j.cell.2017.08.008 PMC5671343

[12] I. Ohad, P. Siekevitz, G. E. Palade, Biogenesis of chloroplast membranes. I. Plastid dedifferentiation in a dark-grown algal mutant (Chlamydomonas reinhardi). J. Cell Biol. **35**, 521–552 (1967). 6064364 10.1083/jcb.35.3.521 PMC2107153

[13] B. D. Engel , Native architecture of the Chlamydomonas chloroplast revealed by in situ cryo-electron tomography. Elife **4**, e04889 (2015). 25584625 10.7554/eLife.04889 PMC4292175

[14] L. C. M. Mackinder , A spatial interactome reveals the protein organization of the algal CO2-concentrating mechanism. Cell **171**, 133–147.e114 (2017). 28938113 10.1016/j.cell.2017.08.044 PMC5616186

[15] Y. Zhan , Pyrenoid functions revealed by proteomics in Chlamydomonas reinhardtii. PLoS One **13**, e0185039 (2018). 29481573 10.1371/journal.pone.0185039 PMC5826530

[16] M. T. Meyer , Assembly of the algal CO_2_-fixing organelle, the pyrenoid, is guided by a Rubisco-binding motif. Sci. Adv. **6**, eabd2408 (2020). 33177094 10.1126/sciadv.abd2408 PMC7673724

[17] A. K. Itakura , A Rubisco-binding protein is required for normal pyrenoid number and starch sheath morphology in Chlamydomonas reinhardtii. Proc. Natl. Acad. Sci. U.S.A. **116**, 18445–18454 (2019). 31455733 10.1073/pnas.1904587116 PMC6744930

[18] C. F. Delwiche, J. D. Palmer, Rampant horizontal transfer and duplication of rubisco genes in eubacteria and plastids. Mol. Biol. Evol. **13**, 873–882 (1996). 8754222 10.1093/oxfordjournals.molbev.a025647

[19] R. Rowan, S. M. Whitney, A. Fowler, D. Yellowlees, Rubisco in marine symbiotic dinoflagellates: Form II enzymes in eukaryotic oxygenic phototrophs encoded by a nuclear multigene family. Plant Cell **8**, 539–553 (1996). 8721755 10.1105/tpc.8.3.539 PMC161119

[20] A. Jenks, S. P. Gibbs, Immunolocalization and distribution of Form II rubisco in the pyrenoid and chloroplast stroma of amphidinium carterae and Form I rubisco in the symbiont-derived plastids of peridinium foliaceum (Dinophyceae). J. Phycol. **36**, 127–138 (2000).

[21] V. Smetacek, Diatoms and the ocean carbon cycle. Protist **150**, 25–32 (1999). 10724516 10.1016/S1434-4610(99)70006-4

[22] Y. Matsuda, B. M. Hopkinson, K. Nakajima, C. L. Dupont, Y. Tsuji, Mechanisms of carbon dioxide acquisition and CO2 sensing in marine diatoms: A gateway to carbon metabolism. Philos. Trans. R. Soc. Lond. B Biol. Sci. **372**, 20160403 (2017). 28717013 10.1098/rstb.2016.0403 PMC5516112

[23] Y. Matsuda, P. G. Kroth, Carbon Fixation in Diatoms in "The Structural Basis of Biological Energy Generation", M. F. Hohmann-Marriott, Ed. (Springer, Netherlands, Dordrecht, 2014), pp. 335–362, 10.1007/978-94-017-8742-0_18.

[24] S. Kikutani , Thylakoid luminal theta-carbonic anhydrase critical for growth and photosynthesis in the marine diatom Phaeodactylum tricornutum. Proc. Natl. Acad. Sci. U.S.A. **113**, 9828–9833 (2016). 27531955 10.1073/pnas.1603112113 PMC5024579

[25] S. Flori , Plastid thylakoid architecture optimizes photosynthesis in diatoms. Nat. Commun. **8**, 15885 (2017). 28631733 10.1038/ncomms15885 PMC5481826

[26] M. Tachibana , Localization of putative carbonic anhydrases in two marine diatoms, Phaeodactylum tricornutum and Thalassiosira pseudonana. Photosynth. Res. **109**, 205–221 (2011). 21365259 10.1007/s11120-011-9634-4

[27] S. Jin , Structural insights into the LCIB protein family reveals a new group of beta-carbonic anhydrases. Proc. Natl. Acad. Sci. U.S.A. **113**, 14716–14721 (2016). 27911826 10.1073/pnas.1616294113 PMC5187666

[28] A. E. Allen , Evolution and functional diversification of fructose bisphosphate aldolase genes in photosynthetic marine diatoms. Mol. Biol. Evol. **29**, 367–379 (2012). 21903677 10.1093/molbev/msr223 PMC3245544

[29] N. Atkinson, Y. Mao, K. X. Chan, A. J. McCormick, Condensation of Rubisco into a proto-pyrenoid in higher plant chloroplasts. Nat. Commun. **11**, 6303 (2020). 33298923 10.1038/s41467-020-20132-0 PMC7726157

[30] J. H. Hennacy, M. C. Jonikas, Prospects for engineering biophysical CO2 concentrating mechanisms into land plants to enhance yields. Annu. Rev. Plant Biol. **71**, 461–485 (2020). 32151155 10.1146/annurev-arplant-081519-040100 PMC7845915

[31] M. F. Liaud, C. Lichtle, K. Apt, W. Martin, R. Cerff, Compartment-specific isoforms of TPI and GAPDH are imported into diatom mitochondria as a fusion protein: Evidence in favor of a mitochondrial origin of the eukaryotic glycolytic pathway. Mol. Biol. Evol. **17**, 213–223 (2000). 10677844 10.1093/oxfordjournals.molbev.a026301

[32] A. A. Hyman, C. A. Weber, F. Julicher, Liquid-liquid phase separation in biology. Annu. Rev. Cell Dev. Biol. **30**, 39–58 (2014). 25288112 10.1146/annurev-cellbio-100913-013325

[33] Y. Shin, C. P. Brangwynne, Liquid phase condensation in cell physiology and disease. Science **357**, eaaf4382 (2017). 28935776 10.1126/science.aaf4382

[34] H. Wang , Rubisco condensate formation by CcmM in beta-carboxysome biogenesis. Nature **566**, 131–135 (2019). 30675061 10.1038/s41586-019-0880-5

[35] T. Wunder, O. Mueller-Cajar, Biomolecular condensates in photosynthesis and metabolism. Curr. Opin. Plant Biol. **58**, 1–7 (2020). 32966943 10.1016/j.pbi.2020.08.006

[36] J. M. Choi, A. S. Holehouse, R. V. Pappu, Physical principles underlying the complex biology of intracellular phase transitions. Annu. Rev. Biophys. **49**, 107–133 (2020), 10.1146/annurev-biophys-121219-081629. 32004090 PMC10715172

[37] W. S. L. Ang, J. A. How, J. B. How, O. Mueller-Cajar, The stickers and spacers of Rubiscondensation: Assembling the heartpiece of biophysical CO2 concentrating mechanisms. J. Exp. Bot. **74**, 612–626 (2022), 10.1093/jxb/erac321. 35903998

[38] A. K. Lancaster, A. Nutter-Upham, S. Lindquist, O. D. King, PLAAC: A web and command-line application to identify proteins with prion-like amino acid composition. Bioinformatics **30**, 2501–2502 (2014). 24825614 10.1093/bioinformatics/btu310 PMC4147883

[39] S. Alberti, A. Gladfelter, T. Mittag, Considerations and challenges in studying liquid-liquid phase separation and biomolecular condensates. Cell **176**, 419–434 (2019). 30682370 10.1016/j.cell.2018.12.035 PMC6445271

[40] B. J. Karas , Designer diatom episomes delivered by bacterial conjugation. Nat. Commun. **6**, 6925 (2015). 25897682 10.1038/ncomms7925 PMC4411287

[41] S. Flori, P. H. Jouneau, G. Finazzi, E. Marechal, D. Falconet, Ultrastructure of the periplastidial compartment of the diatom phaeodactylum tricornutum. Protist **167**, 254–267 (2016). 27179349 10.1016/j.protis.2016.04.001

[42] A. Gruber, G. Rocap, P. G. Kroth, E. V. Armbrust, T. Mock, Plastid proteome prediction for diatoms and other algae with secondary plastids of the red lineage. Plant J. **81**, 519–528 (2015). 25438865 10.1111/tpj.12734 PMC4329603

[43] J. A. Riback , Composition-dependent thermodynamics of intracellular phase separation. Nature **581**, 209–214 (2020). 32405004 10.1038/s41586-020-2256-2 PMC7733533

[44] K. M. Ruff, F. Dar, R. V. Pappu, Ligand effects on phase separation of multivalent macromolecules. Proc. Natl. Acad. Sci. U.S.A. **118**, e2017184118 (2021). 33653957 10.1073/pnas.2017184118 PMC7958451

[45] P. Tompa, N. E. Davey, T. J. Gibson, M. M. Babu, A million peptide motifs for the molecular biologist. Mol. Cell **55**, 161–169 (2014). 25038412 10.1016/j.molcel.2014.05.032

[46] K. Valegård , Structural and functional analyses of Rubisco from arctic diatom species reveal unusual posttranslational modifications. J. Biol. Chem. **293**, 13033–13043 (2018). 29925588 10.1074/jbc.RA118.003518 PMC6109933

[47] J. Wang , A molecular grammar governing the driving forces for phase separation of prion-like RNA binding proteins. Cell **174**, 688–699.e616 (2018). 29961577 10.1016/j.cell.2018.06.006 PMC6063760

[48] F. Luo , Atomic structures of FUS LC domain segments reveal bases for reversible amyloid fibril formation. Nat. Struct. Mol. Biol. **25**, 341–346 (2018). 29610493 10.1038/s41594-018-0050-8

[49] B. Gabryelczyk , Hydrogen bond guidance and aromatic stacking drive liquid-liquid phase separation of intrinsically disordered histidine-rich peptides. Nat. Commun. **10**, 5465 (2019). 31784535 10.1038/s41467-019-13469-8 PMC6884462

[50] E. W. Martin , Valence and patterning of aromatic residues determine the phase behavior of prion-like domains. Science **367**, 694–699 (2020). 32029630 10.1126/science.aaw8653 PMC7297187

[51] M. A. Gebbie , Tuning underwater adhesion with cation-pi interactions. Nat. Chem. **9**, 473–479 (2017). 28430190 10.1038/nchem.2720

[52] S. Kim , Salt triggers the simple coacervation of an underwater adhesive when cations meet aromatic pi electrons in seawater. ACS Nano **11**, 6764–6772 (2017). 28614666 10.1021/acsnano.7b01370

[53] B. Xu , Rigidity enhances a magic-number effect in polymer phase separation. Nat. Commun. **11**, 1561 (2020). 32214099 10.1038/s41467-020-15395-6 PMC7096466

[54] M. C. Ferrolino, D. M. Mitrea, J. R. Michael, R. W. Kriwacki, Compositional adaptability in NPM1-SURF6 scaffolding networks enabled by dynamic switching of phase separation mechanisms. Nat. Commun. **9**, 5064 (2018). 30498217 10.1038/s41467-018-07530-1 PMC6265330

[55] M. -C. Oliver Martin , Data for “A linker protein from a red-type pyrenoid phase separates with Rubisco via oligomerizing sticker motifs”, DR-NTU (Data), 10.21979/N9/IXBAW5, 18 May 2023. PMC10288592 37311001

[56] Z. G. Oh W. Ang S. Bhushan O. Mueller-Cajar. Rubisco from Phaeodactylum tricornutum bound to PYCO1(452-592). Electron Microscopy Data Bank. https://www.ebi.ac.uk/emdb/EMD-33887. 21 July 2022.

[57] Z. G. Oh, W. Ang, S. Bhushan, O. Mueller-Cajar. Rubisco from Phaeodactylum tricornutum bound to PYCO1(452-592). Electron Microscopy Data Bank. https://www.ebi.ac.uk/emdb/EMD-35166. 18 January 2023.

[58] Z. G. Oh, W. Ang, S. Bhushan, O. Mueller-Cajar. PYCO1(452-592) from Phaeodactylum tricornutum. Electron Microscopy Data Bank. https://www.ebi.ac.uk/emdb/EMD-35159. 17 January 2023.

[59] Z. G. Oh, W. Ang, S. Bhushan, O. Mueller-Cajar. Rubisco from Phaeodactylum tricornutum. Electron Microscopy Data Bank. https://www.ebi.ac.uk/emdb/EMD-35158. 17 January 2023.

[60] Z. G. Oh, W. Ang, S. Bhushan, O. Mueller-Cajar. Rubisco from Phaeodactylum tricornutum bound to PYCO1(452-592). wwPDB. https://www.rcsb.org/structure/7Y5K. 21 July 2022

[61] Y. Perez-Riverol , The PRIDE database resources in 2022: a hub for mass spectrometry-based proteomics evidences. Nucleic Acids Research, **50**, D543–D552 (2021). 10.1093/nar/gkab1038. PMC8728295 34723319

[62] Z. G. Oh, O. Mueller-Cajar. A prion-like protein from a red-type pyrenoid forms heterotypic Rubisco condensates via sticker tetrads. PRIDE. https://proteomecentral.proteomexchange.org/cgi/GetDataset?ID=PXD027027. 11 March 2022

