# Supplementary material for: A linker protein from a red-type pyrenoid phase separates with Rubisco via oligomerizing sticker motifs
Source: Proc Natl Acad Sci U S A. 2023 Jun 13;120(25):e2304833120. doi: 10.1073/pnas.2304833120 (PMC10288592; doi:10.1073/pnas.2304833120)
Supplement: Supplementary file 1 — Appendix 01 (PDF) [file pnas.2304833120.sapp.pdf]

Supplementary Information for

A linker protein from a red-type pyrenoid phase separates with Rubisco via oligomerizing sticker motifs

Zhen Guo Oh[1,3,7], Warren Ang[1,4,7], Cheng Wei Poh[1], Soak-Kuan Lai[1], Siu Kwan Sze[1,5], Hoi-Yeung Li[1], Shashi Bhushan[1,2]*, Tobias Wunder[1,6]* and Oliver Mueller-Cajar[1,2,8]*

[1]School of Biological Sciences, Nanyang Technological University, 60 Nanyang Drive, Singapore 637551

[2]Nanyang Institute of Structural Biology, Nanyang Technological University, Singapore 639798

[3]Present address: School of Integrative Plant Science, Cornell University, Ithaca, NY14853, USA

[4]Present address: Boyce Thompson Institute, Ithaca, NY14853, USA

[5]Present address: Health Sciences, Brock University, Niagara Region, 1812 Sir Isaac Brock Way, St. Catharines, ON, Canada, L2S 3A1

[6]Present address: Biozentrum der LMU Muenchen, Dept. Biologie I- Botanik, 82152 Martinsried, Germany

[7]These authors contributed equally

[8]Lead Contact

*Corresponding Authors: sbhushan@ntu.edu.sg (S.B.); tobias.wunder@biologie.uni-muenchen.de (T.W.); cajar@ntu.edu.sg (O.M.-C.)

This PDF file includes:

Supplementary text

Figures S1-S8

Tables S1-S6

Legends for Movies S1-S3

SI References

Materials and Methods

Strains

E. coli DH5α (Thermo Fisher Scientific) were used for the amplification of plasmid constructs. Subsequently, clones were selected and cultivated in LB liquid media with the respective antibiotics at 37°C and grown overnight. BL21 (DE3) (Sigma-Aldrich) were used for recombinant protein production, culturing conditions for protein expression are provided in 'Method Details'.

The sequenced strain, *Phaeodactylum tricornutum* Pt 1 8.6, CCMP 2561 from Provasoli-Guillard National Center for Marine Algae and Microbiota, Bigelow Laboratories for Ocean Sciences was used in this study (1). Solid and liquid cultures were propagated in modified F/2 media (2, 3), where seawater is replaced with 16.6 g dissolved artificial sea salt (Tropic Marin® Sea Salt CLASSIC), at room temperature and in continuous illumination of ~100 μmol m^{-2} s^{-1}.

Plasmids and proteins

The oligonucleotides used in this study are listed in Table S5. Selected protein sequences encoded by the plasmids used in this study are shown in Table S6.

Genomic DNA

5 mL of mid-log *P. tricornutum* culture were pelleted by centrifugation (16,000 x g/15 min/4 °C). The cell pellet was washed twice with 375 mM Sorbitol and resuspended in 0.4 mL 100% Edwards Solution (4) (200 mM Tris-HCl pH 8.0, 250 mM NaCl, 25 mM EDTA, 0.5% (w/v) SDS) and cell lysis was performed through heating (65 °C/5 min). The lysate suspension was centrifuged (16,000 x g/15 min/4 °C). To precipitate DNA, 0.4 mL isopropanol was added to the supernatant and incubated on ice for 30 min. The suspension was centrifuged (16,000 x g/1 hr/4 °C). The DNA pellet was washed with 0.5 mL 70% ethanol and the suspension was centrifuged (16,000 x g/15 min/4 °C). The pellet was placed under the fume hood for drying and evaporation of trace ethanol. The genomic DNA pellet was redissolved with autoclaved Milli-Q water.

Plasmids

PYCO1 (Phatr3_J49957) was amplified from genomic DNA using primers 1 and 2, ligated into pGem-T Easy (Promega), and then sequenced to yield pGemT*PYCO1*. For protein expression, the first 30 N-terminal amino acids were excluded from any PCR amplifications as they were predicted to contain the chloroplast transit peptide. *PYCO1(31-592)* was amplified with flanking *Eco*RI and *Hind*III restriction sites using primers 3 and 4 and ligated between the *Eco*RI and *Hind*III sites of pHueFLAG (5). An *Nhe*I restriction site was introduced 5' of the FLAG-epitope encoding sequence using site directed mutagenesis utilizing primers 5 and 6. *FLAG-PYCO1* was subsequently ligated between the *Nhe*I and *Hind*III sites of pET-24b (Novagen) to yield pET24b*FLAGPYCO1ΔN30His6*.

PtCA1 (*Phatr3_J51305*) amplified from genomic DNA using primers 37 and 38 was introduced into pDEST-C-ECFP regulated by FcpB promoter and FcpA terminator (6). This expression cassette was flanked with *Sph*I restriction sites by amplification using primers 7 and 8, and ligated into pGemT-Easy to yield pGemT*PtCA1ECFPSphI*. *PYCO1* was amplified from pGemT*PYCO1* using primers Oligo 9 and Oligo 10, which introduced flanking *Cla*I and *Asc*I restriction sites. *PtCA1* in pGemT*PtCA1ECFPSphI* was excised using *Cla*I and *Asc*I and the corresponding *PYCO1* fragment was inserted. Finally, the expression cassette was released using *Sph*I and introduced into the *Sph*I site of pPtPuc3 (7) to give pPtPuc3_*FcpB_PYCO1ECFP_FcpA*.

pTrc*RsLS* was constructed by amplifying the *R. sphaeroides* Rubisco operon from pET30b*RscbbLS* (8) using primers 39 and 40 and inserted between the *Nco*I and *Hind*III restriction sites of pTrcHisB to generate pTrc*RsLS*. An *Nde*I site was introduced into the intergenic region using primers 41 and 42. The sequence encoding the *P. tricornutum* Rubisco small subunit (*PtrbcS*, YP_874417.1) was amplified from genomic DNA with flanking *Nde*I and *Hind*III sites using primers 43 and 44. The *PtrbcS* gene was then inserted between the *Nde*I and *Hind*III sites of the modified pTrc*RsLS* vector to give pTrc-*RsLPtS*.

The gene encoding monomeric enhanced fluorescent protein (mEGFP) was amplified from pHue*FLAGCrEPYC1GFP* (9) using primers 73 and 74, which inserted flanking *Sal*I and *Not*I restriction sites. The gene was then inserted between *Sal*I and *Not*I sites of pHue (10) to yield pHue*mEGFP*.

To construct PYCO1-GFP, mEGFP was amplified from pHue*FLAGCrEPYC1GFP* (9) using primers 11 and 12 to insert flanking *Hind*III and *Not*I sites. mEGFP was inserted on the C-terminus of PYCO1 in pET24b*FLAGPYCO1ΔN30His6* using said restriction sites to produce pET24b*FLAGPYCO1ΔN30GFPHis6*. For PYCO1-mRuby, pUC57*mRuby3* (Genscript) containing mRuby3 with flanking *Hind*III and *Not*I restriction sites were excised and replaced mEGFP from pET24b*FLAGPYCO1ΔN30GFPHis6* to yield pET24b*FLAGPYCO1ΔN30mRubyHis6*.

PYCO1(452-592) and PYCO1(483-592) was amplified from pET24b*FLAGPYCO1ΔN30His6* using primers 13 and 14, or 17 and 18 respectively. The amplicon flanked the genes with *EcoR*I and *Hind*III restriction sites. These amplicons were subsequently used to replace full length PYCO1 from pET24b*FLAGPYCO1ΔN30His6* using said restriction enzymes to produce pET24b*FLAGPYCO1(452-592)His6* and pET24b*FLAGPYCO1(483-592)His6*.

Deletion of "KWSPR" motif on PYCO1(452-592) was done through inverse PCR mutagenesis on pET24b*FLAGPYCO1(452-592)His6* with primers 15 and 16. The amplicons were joined head-to-tail using blunt-end ligation to produce pET24b*FLAGPYCO1(452-592)ΔKWSPRHis6*. Mutation of specific residues on the "KWSPR" motif on PYCO1(452-592) was conducted using site-directed mutagenesis with primers 19 and 20, 21 and 22, 23 and 24, or 25 and 26 to produce pET24b*FLAGPYCO1(452-592)K475AHis6*, pET24b*FLAGPYCO1(452-592)W476AHis6*, pET24b*FLAGPYCO1(452-592)R479AHis6*, and pET24b*FLAGPYCO1(452-592)R479QHis6* respectively.

Deletion of the FLAG motif on pET24b*FLAGPYCO1ΔN30His6* was conducted using inverse PCR mutagenesis with primers 39 and 40. The amplicons were joined head-to-tail using blunt-end ligation to produce pET24b*PYCO1ΔN30His6*. To construct PYCO1 Y→A, W→A and W/Y→A, pUC57*PYCO1YtA*, pUC57*PYCO1WtA*, and pUC57*PYCO1WYtA* were synthesized by Genscript which flanked these genes with *EcoR*I and *Hind*III restriction sites. These genes replaced PYCO1 on pET24b*PYCO1ΔN30His6* to produce pET24b*PYCO1ΔN30His6YtA*, pET24b*PYCO1ΔN30His6WtA*, and pET24b*PYCO1ΔN30His6W/YtA* respectively.

Protein expression and purification

Proteins were purified using appropriate chromatography columns connected to an Akta Purifier system (Cytiva). Purified proteins were buffer exchanged (Micro Bio-Spin® Columns with Bio-Gel® P-6) or dialyzed (Slide-A-Lyzer™ MINI Dialysis Device, 2K MWCO; Thermo Fischer Scientific) into relevant assay buffers before use. Protein concentrations were determined spectrophotometrically at 280 nm using a NanoDrop™ One (Thermo Fischer Scientific) instrument.

PYCO1, PYCO1-GFP, PYCO1-mRuby, truncations and mutants

PYCO1, PYCO1-GFP, PYCO1-mRuby, as well as truncations and mutants were produced and purified from E. coli BL21 (DE3) cells harbouring the relevant plasmids. Briefly, 1 L LB was inoculated with overnight grown starter cultures and incubated (37 °C/220 rpm) until OD_{600} ~0.6. 1 mM IPTG was added to induce protein production (37 °C/220 rpm) for 4 hrs and cells were harvested. With the exception of fluorescently tagged PYCO1: PYCO1-GFP and PYCO1-mRuby, cell biomass was resuspended in low imidazole buffer (20 mM Tris-HCl, pH 8.0, 250 mM NaCl, 10 mM imidazole, 12.5% (v/v) glycerol) supplemented with 1 protease inhibitor tablet, 3 mM β -mercaptoethanol and 1 mM phenylmethylsulfonyl fluoride (PMSF). Cell suspensions were boiled for 20 min with gentle agitation at every 5 min interval. After boiling, the suspension was left to cool on ice for 15 min before sonication (15 secs burst, 30 secs recovery, 30% amplitude). Centrifugation was performed (13,000 x g/20 min/20 °C) to clarify the lysate.

The clarified lysates of cells producing PYCO1 fragments or PYCO1(W/Y→A) were filtered and loaded onto an immobilized metal affinity chromatography column (HisTrap™; Cytiva), equilibrated with low imidazole buffer and bound proteins were eluted using a linear imidazole gradient to 0.4 M imidazole buffer. Fractions were pooled and loaded on a size-exclusion chromatography column (Superdex HiLoad™ 16/600 200 pg; Cytiva) equilibrated in 20 mM Tris-HCl, pH 8.0, 250 mM NaCl, 12.5% (v/v) glycerol. Fractions containing the target proteins were concentrated with appropriate pore-sized centrifugal filters, aliquoted and flash frozen in liquid N_2.

PYCO1-GFP and PYCO1-mRuby biomass and pelleted cell debris of PYCO1, PYCO1(Y→A) and PYCO1(W→A)were resuspended in low imidazole urea buffer (20 mM Tris-HCl, pH 8.0, 250 mM NaCl, 10 mM Imidazole, 8 M Urea) with a Potter-Elvehjem grinding chamber. The suspension was clarified with centrifugation (13,000 x g/45 min/20 °C) and the resulting supernatant was filtered and loaded onto an immobilized metal affinity chromatography column as described above, equilibrated with low imidazole urea buffer and bound proteins were eluted with a linear imidazole gradient to 0.4 M imidazole urea buffer. Fractions were pooled and loaded on a size-exclusion chromatography column as above, equilibrated in 20 mM Tris-HCl, pH 8.0, 250 mM NaCl,8 M Urea. Protein containing fractions were concentrated and stored as described above.

***Rs*Rubisco, *Rs*L*Pt*S**

Proteobacterial rubisco and chimeric rubisco were expressed and purified from *E. coli* BL21 (DE3) cells harbouring pTrc*RsLS* and pTrc*RsLPtS* respectively. 1 L LB was inoculated with overnight grown starter cultures and incubated (37 °C/220 rpm) until OD_{600} ~0.6 and cell cultures were equilibrated to 23 °C (~30 min). 0.5 mM IPTG was added to induce protein production (37 °C/220 rpm/overnight). Cells were harvested and incubated in buffer A (20 mM Tris-HCl, pH 8.0, 50 mM NaCl, 1 mM EDTA, 12.5% (v/v) glycerol) containing 0.3 mg/mL lysozyme for 30 minutes on ice prior to adding 1 mM PMSF and sonication (15 secs burst, 30 secs recovery, 30% amplitude). Centrifugation was performed (20,000 rpm/45 min/4 °C) to clarify the lysate. Supernatant was filtered and loaded onto an anion exchange chromatography column (HiScale 26/20 column packed with Source30Q anion exchange resin; Cytiva) equilibrated with buffer A and bound proteins were eluted with a linear salt gradient to 0.5 M NaCl. Fractions were pooled and loaded on a size-exclusion chromatography column as above, equilibrated in buffer A. Protein containing fractions were concentrated and stored as described above.

***Pt*Rubisco Purification**

*Pt*Rubisco was purified from 12 L cultures of mid logarithmic *P. tricornutum* grown in F/2 media containing 30 μg/mL Kanamycin under airlift conditions. Cells were centrifuged (6000 rpm/30 min/4

°C) and resuspended in reduced buffer A (20 mM Tris-HCl pH 8.0, 50 mM NaCl, 1 mM EDTA, 12.5% (v/v) glycerol, 10 mM DTT) supplemented with 4 complete protease inhibitor cocktail (Roche), 1 mM PMSF. Cells were lysed using a Microfluidizer LM20 (20,000 psi/5 cycles) and additional buffer was added to dilute the lysate to 240 mL. Centrifugation was performed (20,000 rpm/1 hr/4 °C) to clarify the lysate. Supernatant was filtered and loaded onto an anion exchange chromatography column as described above, equilibrated with reduced buffer A and bound proteins were eluted with a linear salt gradient to 0.5 M NaCl. Additionally, 50 µL 1 M DTT was added to each 10 mL Rubisco containing fractions and kept in 4 °C. The next day, these fractions were pooled and loaded on a size-exclusion chromatography column as above, equilibrated in reduced buffer A. Additional, 12.5 µL 1 M DTT was added to each 2.5 mL Rubisco containing fractions and kept in 4 °C.The following day, these fractions were pooled and loaded on an anion exchange chromatography column (MonoQ 10/100; Cytiva), equilibrated with reduced buffer A and bound proteins were eluted with a linear salt gradient to 0.5 M NaCl. Protein containing fractions were concentrated and stored as described above.

Purification of other Rubiscos

Arabidopsis thaliana Rubisco was purified from rosettes as described (11). *Chlamydomonas reinhardtii* Rubisco was purified from strain CC2677 (Chlamydomonas Resource centre) as described (9). *Acidthiobacillus ferrooxidans* Form II Rubisco was purified from *Escherichia coli* harbouring pHue*AfcbbM* as described (5).

Atto594 labelling of *Pt*Rubisco

Primary amines of *Pt*Rubisco were labelled using Atto 594 NHS Ester (Atto 594 Protein Labeling Kit, Sigma-Aldrich, #68616) following the manufacturer's instructions. The dye-labelled protein was applied to an analytical size exclusion chromatography column (Superdex 200 increase 3.2/30 GL; Cytiva) equilibrated with storage buffer. The Rubisco containing fractions were concentrated and stored as described above. Approximately two dye molecules were bound per Rubisco holoenzyme.

Conjugation of *P. tricornutum*

P. tricornutum expressing PYCO1-CFP was generated using bacterial conjugation (7, 12). Overnight starter culture of *E. coli* DH10B pTA-Mob transformed with *pPtPuc3-FcpB Pro-PYCO1-CFP-FcpA Ter* was inoculated into 100 mL LB and was grown at 37 °C, 200 rpm until OD600 ~0.8-1.0. Cells were harvested by centrifugation (3, 000 x g/10 min/4 °C) and resuspended in 1 mL LB media. In parallel, four-day old *P. tricornutum* solid cultures were scraped with liquid F/2 media and cell concentration was adjusted to ~5.0 x 10^8 mL^{-1}. Equal volumes of *P. tricornutum* and *E. coli* cell suspensions was mixed, plated on F/2, 5% LB media plates and incubated in dark at 30 °C for 90 min. The plates were shifted to room temperature and were grown under continuous light conditions. After 2 days, F/2 media was added to the plate and the lawn of cells were scraped and transferred to F/2 media plates supplemented with 50 µg/mL zeocin. Colonies emerged 10 to 14 days later and were subcultured in either liquid or solid F/2 media.

One-week old liquid cultures were screened using epifluorescence microscopy and laser scanning confocal microscopy.

Immunoprecipitation and Mass spectrometry

A polyclonal antibody was raised in rabbits against the Rubisco large subunit peptide RYESGVIPYAKMC and affinity purified (Singapore Advanced Biologics). Protein A resin (Invitrogen™ Protein A - Sepharose™ 4B) was aliquoted into 1.5 mL eppendorf tubes, and washed twice with Co-IP buffer (20 mM Tris-HCl pH 8.0, 50 mM NaCl, 0.1 mM EDTA, 12.5% v/v glycerol) by centrifugation (8,200 x g/1 min/4 °C). Next, the resin was incubated with 9.2 µg/mL anti-PtRubisco on a rotator (2 hr/4 °C) and washed twice with Co-IP buffer. A blocking step was performed by incubating the resin with 2 mg/mL BSA on the rotator (1 hr/4 °C) and washed twice with Co-IP buffer.

In the absence of cross-linking, 50 mL cultures of mid-logarithmic *P. tricornutum* were lysed using glass beads, 150-212 µm (Sigma-Aldrich, G1145) in 167 µL Co-IP buffer supplemented with 1 mM PMSF, 5 mM DTT and recovered with 1.5 mL lysis buffer. The recovered lysate was clarified by centrifugation (14,800 rpm/20 min/4 °C). The soluble fraction was then added to anti-PtN Rubisco

bound resin and incubated on a rotator (3 hr/4 °C) and washed twice with Co-IP buffer. Protein elution was carried out by adding 2.5x SDS loading buffer and the sample was boiled (95 °C/5 min) and centrifuged (17,600 x g/1 min) for analysis by 12.5% SDS-PAGE.

In three experiments dithiobis(succinimidyl propionate (DSP, Thermo Fischer Scientific) was included to capture transient protein-protein interactions. 50 mL cultures of *P. tricornutum* was resuspended in 167 µL Co-IP XL buffer (20 mM HEPES pH 8.0, 50 mM NaCl, 0.1 mM EDTA, 12.5% glycerol) and incubated with 4 mM DSP (30 min/room temperature). The crosslinker was then quenched by adding Tris-HCl pH 8.0 to 50 mM. The cells were then lysed and processed as described above.

SDS samples were prepared for mass spectrometry by concentrating samples on a 12% separating layer through a homemade blocking SDS gel: consisting of 4% stacking gel, 12% separating gel and 25% blocking gel. After gel electrophoresis, the gel was stained, and the separating layer containing the proteins was excised. Proteomic analysis of the samples was performed by the NTU-SBS Proteomics and Mass Spectrometry Service. Digestion of the separating layer was performed as described (13) with some modifications. Briefly, after in-gel digestion with trypsin, 50 µL of 50% acetonitrile (ACN)/5% formic acid (FA) was added to the gel pieces and sonicated for 30 min. This extraction procedure was repeated three times, and ∼150 µL of extract was collected. Extracts were pooled and concentrated to dry using an SPD 2010 SpeedVac system (Thermo Electron, Waltham, MA). 0.1% FA in HPLC water was added to a final volume of 30 µL for direct LC-MS/MS analysis.

The peptides were separated and analyzed using a Dionex Ultimate 3000 RSLCnano system coupled to a Q Exactive instrument (Thermo Fisher Scientific, MA, USA). Separation was performed on a Dionex EASY-Spray 75 μm × 10 cm column packed with PepMap C18 3 μm, 100 Å (Thermo Fisher Scientific) using solvent A (0.1% formic acid) and solvent B (0.1% formic acid in 100% ACN) at a flow rate of 300 nL/min with a 60 min gradient. Peptides were then analyzed on a Q Exactive apparatus with an EASY nanospray source (Thermo Fisher Scientific) at an electrospray potential of 1.5 kV. Raw data files were processed and searched using Proteome Discoverer 2.1 (Thermo Fisher Scientific). The Mascot algorithm was then used for tandem mass spectra searching to identify proteins referenced to both the *P. tricornutum* nuclear genome sequence database (1) and SwissProt.

Mass spectrometry data was sorted accordingly to the predicted localization of nuclear encoded *P. tricornutum* proteins using Signal P4.1 (14) and ASAFind (15). The Swissprot database contains a mixture of nuclear and chloroplast encoded proteins from *P. tricornutum*. To clarify the gene locus, Swissprot hits were further referenced to the *P. tricornutum* EnsemblProtist database (16) using a BLAST search. The mass spectrometry proteomics data have been deposited to the ProteomeXchange Consortium via the PRIDE (https://www.ncbi.nlm.nih.gov/pmc/articles/PMC8728295/) partner repository with the dataset identifier PXD027027.

Bioinformatic analysis of PYCO1

Distribution of net charge per residue (NCPR) of PYCO1 was analyzed using CIDER (http://pappulab.wustl.edu/CIDER/) (17). The disorder prediction of PYCO1 was performed with the PONDR algorithm (VLXT) (18) (http://www.pondr.com), the bold line represents disordered regions. The hydropathy plot of PYCO1 was analyzed with a sliding window size of 9 (19) (https://web.expasy.org/protscale/). Prion like domains of PYCO1 was predicted with Prion Like Amino Acid Composition (PLAAC) algorithm (20) (http://plaac.wi.mit.edu/), with a minimum length of 60 amino acids and default background frequencies, predicted prion like domains are denoted with a red line.

Light microscopy

Chlorophyll visualization of diatoms and localization of the fluorescently tagged proteins was conducted using a Nikon Inverted Ti microscope. 5 µL liquid algal cultures were loaded onto microscope slides. Chlorophyll *a* auto fluorescence was detected using Cy5 filter settings, while CFP was detected using FITC filter settings.

Super-resolution images were captured with a Zeiss LSM710 confocal microscope equipped with an Airyscan detector using a Plan-Apochromat 100x/1.46 oil objective. Images were post-processed using Zen Blue (Carl Zeiss). The CFP signal was excited at 405 nm and chlorophyll fluorescence at 633 nm.

Brightfield images were acquired using T-PMT through 488 nm laser line. Reconstruction of chloroplast and CFP was conducted using stacks opened in Fiji (21).

PYCO1 or PYCO1-Rubisco condensates were prepared in 5 or 10 µL volumes and placed on a Nikon Interted Ti microscope. DIC images were captured using DIC filter settings, GFP was detected using FITC filter settings and mRuby was detected using mCherry filter settings.

Fluorescence recovery after photobleaching

Condensate and diatom FRAP assays presented in Figures 1 and 2 were performed using Zen 2.3 SP1 (black) software on a Zeiss LSM710 confocal microscope equipped with a 1.46 numerical aperture (NA) plan-Apo x100 oil immersion lens (Carl Zeiss, Germany) at room temperature. Each FRAP experiment started with 5 control scans before bleaching the region of interest (ROI) by exposing the ROI 50 times at 75% intensity of a 488 nm wavelength argon laser GFP-labelled. The photobleached region was then allowed to recover for 75 cycles, with laser power attenuated to 5% intensity for both 488 nm laser. FRAP experiments settings for diatoms was similar as described above. A square ROI with a pixel of 4 (0.5 µm) was exposed 100 times at 100% intensity of the 458 nm argon laser and 405 nm laser, before allowing to recover for 75 cycles with laser power attenuated to 4.5% for the 458 nm laser.

Bleaching of condensates presented in Fig. 4 and Fig. S5D were carried out using a Nikon Inverted Ti2 confocal microscope equipped with a 1.46 NA x100 oil immersion lens at room temperature (Image acquisition control: MetaMorph, Molecular Devices). Samples were mixed and immediately placed onto the microscope slides. Imaging was initiated after approximately two minutes, and data capture was completed within seven minutes. Laser wavelength at 488 nm was used to image (5% power) and bleach (70% power for10 iterations) GFP-tagged PYCO1, while 561 nm was used to image (5% laser power) and bleach (100% power for 80 iterations) Atto-594 labelled rubisco. Condensates were imaged for 2 seconds at 500 ms interval before being bleached. Upon bleaching, the fluorescence intensity was allowed to recover for 1 minute where images were captured every 1 second. In Fig. 4B the bleached condensates were allowed to recover for 30 minutes and images were captured every 15 seconds.

Sedimentation Assays

In all LLPS assays, the protein components were first buffer exchanged into 20 mM Tris-HCl, pH 8.0. Reactions were performed in 5-10 µL volumes.

In homotypic LLPS reactions the protein was diluted using 20 mM Tris-HCl pH8.0. NaCl solution (5 M) was added to achieve the appropriate final concentration to induce phase separation.

The mixture was incubated for 8 min at room temperature and subsequently centrifuged (14,800 rpm/3 min/4 °C) to sediment the condensates in a pellet fraction. The supernatant fraction was carefully separated and 5X SDS loading buffer was added to the supernatant accordingly. Pellet fractions were resuspended in 2.5X SDS loading buffer. Samples were boiled (95 °C/5 min) and centrifuged (17,600 x g/1 min) prior to analysis on SDS-PAGE.

For heterotypic PYCO1-Rubisco LLPS, Rubisco was first diluted in 20 mM Tris-HCl pH8.0. NaCl solution (5M) was then added to give a final concentration of 150 mM unless indicated otherwise. PYCO1 was added last. Incubation, centrifugation and analysis were carried out as described above.

The sample loading volume was adjusted to ensure between 1-5 µg of Rubisco were applied for each experiment to allow densitometric analysis to be performed.

Densitometry was used to quantify the relative amount of proteins found in supernatant and pellet fractions of sedimentation assays. Coomassie Blue stained gels were scanned using a GS-800TM Calibrated Densitometer (Bio Rad) and analyzed using Quantity One (Bio-Rad). Lanes to be quantified were manually framed prior to semi-automatic detection of protein bands. Lane-based rolling disk background subtraction was performed. The band volume (average OD of the band times its area, INT x mm^2) of Rubisco large subunit and PYCO1 bands was determined. The volume of the respective pellet band was divided by the sum of the band volumes of supernatant and pellet to calculate the proportion of protein sedimented in the experiment.

Homotypic PYCO1 condensate reversibility Assays

After performing the sedimentation with PYCO1, as described above, reversibility of PYCO1

condensates was probed by adding either buffer (20 mM Tris-HCl or 20 mM Tris-HCl, 150 mM NaCl) to the pellet fraction. The mixture was incubated (5 min/room temperature) and subjected to centrifugation (14,800 rpm/2 min/4 °C). Supernatant and pellet fractions were analyzed as described above.

Turbidity Assays

PYCO1 condensates were prepared in 10 µL volumes as described under sedimentation assays. The mixture was incubated for 5 minutes at room temperature and the absorbance at 340 nm was recorded using a NanoDrop One spectrophotometer (Thermo Fisher Scientific). The concentration at which absorbance readings increased sharply was taken as the saturation concentration (C_{sat}) for PYCO1.

Quantification of light and dense phase concentrations

C_{dense} and C_{light} were obtained using quantitative fluorescence confocal microscopy using an LSM710 (Zeiss). A standard curve was constructed using known concentrations of purified GFP by diluting GFP to concentrations ranging from 2.5 to 20 µM in 20 mM Tris-HCl pH 8.0 and 50 mM NaCl. Imaging for all solutions was done using the following parameters: excitation laser at 488 nm using 2.5% intensity, pinhole set to 0.47 airy units, and master gain set to 800. 20 µM GFP was first imaged to ensure that the detector was still within the linear range (below 150 intensity units for an 8-bit image) at the highest possible concentration of GFP. Images of GFP solution were captured for 10 Z-stack slices at intervals of 0.26 µm. Three independent dilutions of each GFP concentration was prepared, imaged, and averaged to construct the standard curve. The graph for intensity against GFP concentration was fitted as a linear function $y = ax + b$.

PYCO1 homotypic condensates were prepared in 5 µL volumes with each sample consisting of 98% PYCO1 and 2% PYCO1-GFP corresponding to a total of 8.6 µM protein in a buffer of 20 mM Tris-HCl pH 8.0 and 150 mM NaCl. For heterotypic condensates, 0.17 µM of PYCO1-GFP and 2 µM of PYCO1 were mixed with the corresponding concentrations of PtRubisco. Imaging of the condensates occurred after 2 min to allow surface-attachment coarsening of the micro-condensates. A total of 12 to 15 Z-stack slices were imaged starting from below the glass slide in intervals of 0.26 µm to fully

cover the height of the condensates. Three independent samples were prepared and imaged in the same way. Since different laser batches and laser lifetime significantly influences the reading of this experiment, all images were collected within a period of two weeks to prevent influences from hardware variation.

Images were processed using Fiji. A region of interest was placed on the images covering only the middle section of the condensates to acquire C_{dense}. Multi-measure mode was adopted to obtain a reading of all 12 to 15 slices, where the slice with the maximum intensity reading was used to estimate C_{dense}. To acquire C_{light}, the region of interest was placed on the bulk solution instead, and intensity was selected from the last 3 slices within the sample. The Partition coefficient could be calculated from C_{dense} and C_{light} (Equation 2).

Partition Coefficient P = C_{dense}/C_{light} (Equation 2)

An indirect approach was utilized to estimate the concentration of *Pt*Rubisco in heterotypic condensates. In brief, this involves deriving the values of PYCO1 C_{dense} as described above and sedimentation-densitometry data to calculate the mass of PYCO1 and *Pt*Rubisco in pellet fractions to derive a volume fraction to the dense phase (Equation 3). We assume that *Pt*Rubisco and PYCO1 must exist in the same dense phase. As such, the estimation *Pt*Rubisco C_{dense} can be approximated using the Equation 3 shown below.

$$\frac{Mass\ of\ PtRubisco\ in\ pellet}{Concentration\ \left(\frac{\mu g}{\mu L}\right)\ of\ PtRubisco} = Volume\ fraction\ of\ condensate = \frac{Mass\ of\ PYCO1\ in\ pellet}{Concentration\ (\frac{\mu g}{\mu L})\ of\ PYCO1}$$

(Equation 3)

Radiometric Rubisco Activity assays

$^{14}CO_2$-fixation assays were performed in 10 µL reaction volumes at 25 °C in 2 mL Eppendorf tubes containing final concentrations of 32 µM Rubisco active sites with or without 2 µM PYCO1 in 20 mM Tris-HCl, pH 8,0, 110 mM NaCl, 10 mM MgCl, 5 mM DTT, 4 mM RuBP and 20 mM

$NaH^{14}CO_3$ (20 Bq $nmol^{-1}$). Briefly, 41.6 µM Rubisco active sites were activated for 1 hr in the absence of PYCO1 and RuBP. PYCO1 (20 µM stock) in 20 mM Tris-HCl pH 8.0 was added to trigger LLPS of activated Rubiscos and reactions allowed to incubate (2 min at room temperature). Assays were initiated by adding 1.3 µL of 31 mM RuBP to a final concentration of 4 mM. 20 µL 50% (v/v) formic acid was added to stop reactions at 20 s and 40 s timepoints. The specific activity of $^{14}CO_2$ was measured using 4 nanomoles of RuBP. This reaction was assayed for 1 hr before addition of formic acid as described above. Samples were dried with a heat block and resuspended in 500 µl water supplemented with 1 mL Ultima Gold XR scintillant before quantification using a scintillation counter.

Controls were performed accordingly: for the positive control in place of 20 µM PYCO1 in 20 mM Tris-HCl pH 8.0, 20 mM Tris-HCl pH 8.0 was added while for negative controls RuBP was substituted with 3 mM HCl. Samples without radioactive labels were also analyzed with the Nikon Inverted Ti microscope and sedimentation assays to validate phase separation.

Native-PAGE gel shift assays

PYCO1 protein (and its variants) were added to 0.18 - 0.36 µM Rubisco in 20 mM Tris-HCl, pH 8.0, 50 mM NaCl. The sample was analyzed using 6% Native PAGE. A total of 2 µg Rubisco was loaded per well.

Cryo-EM single particle analysis and image processing

300 mesh Quantifoil 2/2 copper grids with 2 nm carbon support (Electron Microscopy Sciences) were glow-discharged for 15 seconds. Samples containing 0.9 µM of Rubisco with 21.6 µM of PYCO1(452-592) were mixed in 20 mM Tris-HCl pH 8.0 and 50 mM NaCl in 5 µL volumes. The sample chamber of the Vitrobot Mark IV (FEI Company) was maintained at 4 ºC with 100% relative humidity. 4 µL of sample was applied onto the grids and blotted once after 30 seconds (blot force of 1 for 2.5 seconds). The grids were then plunge-frozen into liquid ethane. The grids were screened on

Tecnai Arctica 200 kV transmission electron microscope (FEI Company) for appropriate sample quality and ice thickness.

Grids were loaded onto an FEI 300 kV Titan Krios transmission electron microscope (FEI Company) at National University of Singapore (NUS) equipped with a Gatan K3 camera. Full automatic data acquisition was conducted using SerialEM. Movies were captured at 105,000X magnification corresponding to a pixel size of 0.858 Å. Dose rate was set to a total of 65 electrons collected over 50 movie frames where each frame received a dose of 1.3 electrons per pixel. Spherical aberration constant of the objective lens was 2.7 mm with an objective aperture of 100 µm. The dataset was imaged at a defocus range between -0.8 to -1.6 µm. A total of 8,861 movies were collected in the dataset.

Movies were imported into Relion 3.1 (22), binned 2x and motion corrected using MotionCor2 (23). CTF was estimated using CTFFind4.1 (24). A total of 3,354,751 particles were picked using laplacian of gaussion and extracted from the micrographs. These particles were subjected to several rounds of reference-free 2D and 3D classification to remove bad particles. This gave a final number of 256,796 good particles to work with. 3D refinement using these particles yielded a density map at 2.2 Å at C1 symmetry and 2.1 Å at D4 symmetry. To resolve PYCO1 density on the structure, a mask was placed over the central pore of Rubisco and processed using multibody refinement. This produced a density map at 2.6 Å resolution representing a short segment of PYCO1. The two density maps were combined and saved as a single map file.

Model building, fitting, and refinement

The model of Rubisco from *Thalassiosira antarctica* (25) (PDB ID: 5mz2) was placed into the density map. The amino acid sequence was changed to *P. tricornutum* using CHAINSAW (26) and was used as rigid body docking for our Rubisco. Real space refinement was conducted using Phenix (27) to produce a model of Rubisco from *P. tricornutum*. PTMs and flexible fitting were modified in COOT (28) by manually going through the large and small subunit of Rubisco. Additional real space refinement was conducted in COOT. The full-length sequence of PYCO1 was placed into AlphaFold2 (29) to predict possible secondary structures. C-terminal residues “GNLASEWASMNT” and

“AAEWGSMNQ” were both predicted to contain alpha helices. These helices were placed into the additional density at the LSU of Rubisco, flexible-fitted, and real space refined in COOT, where the density map favoured the latter sequence. On the small subunit of Rubisco, a peptide with the sequence “KWSPRGGS” was built manually, and flexible-fitted into the density map in COOT. Figures from the manuscript were produced using UCSF Chimera (30).

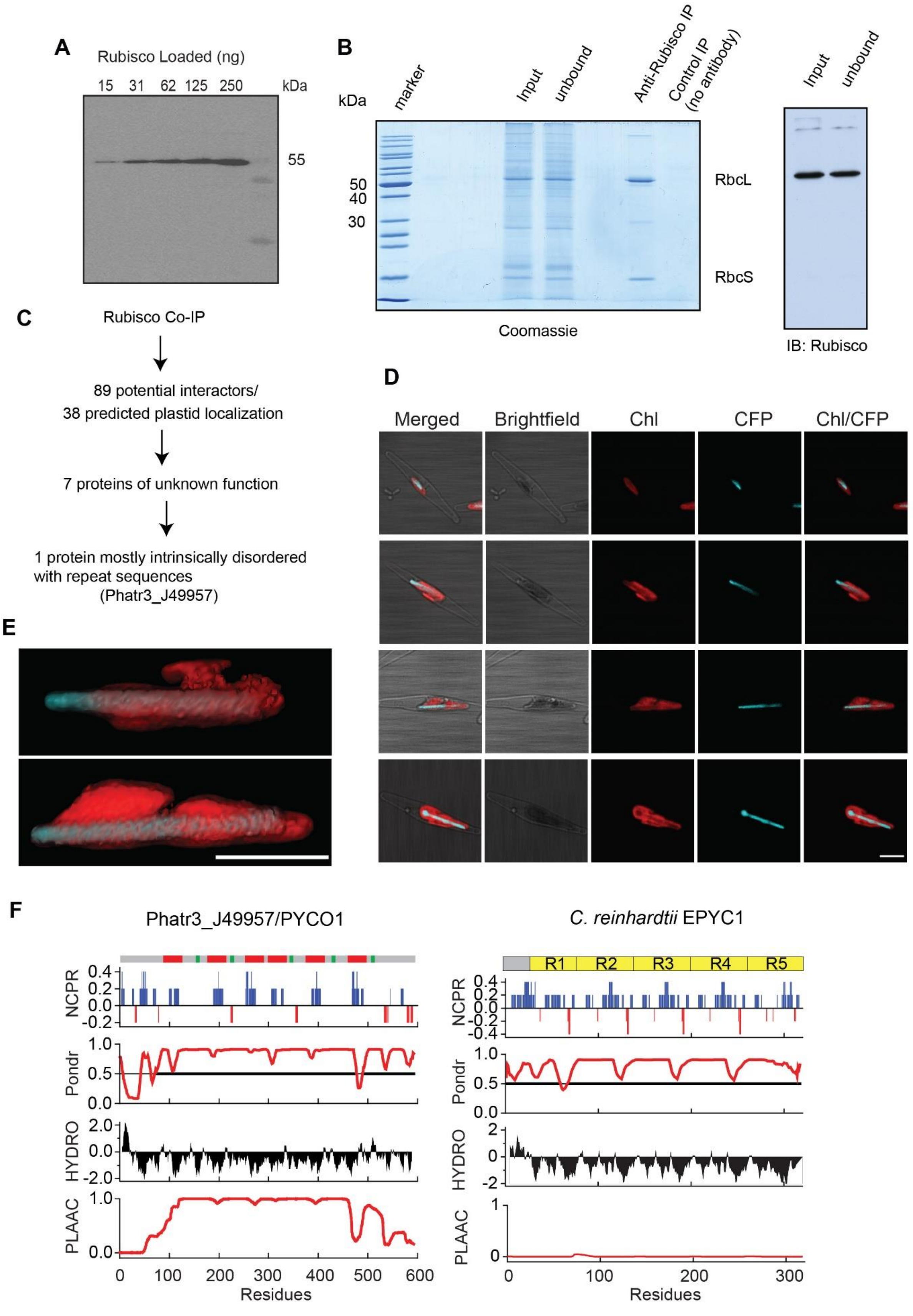

Fig. S1. Co-immunoprecipitation of PtRubisco identifies PYCO1

(A) Evaluation of the antibody used for immunoprecipitation. The indicated mass of purified diatom Rubisco was separated by SDS-PAGE and an immunoblot performed. (B) SDS-PAGE analysis of samples derived from a Rubisco immunoprecipitation experiment (Dataset S1, IP Rbc1). The immunoblot demonstrates the specificity of the antibody against the algal lysate. (C) Bioinformatic evaluation of putative Rubisco interactors leads to identification of PYCO1 (Phatr3_J49557). (D) Confocal microscopy images of multiple cells expressing PYCO1-CFP. Scale bar, 5 µm. (E) Additional 3D reconstructions of confocal sections. Scale bar, 5 µm. (F) Comparative bioinformatic analysis of the amino acid sequences of PYCO1 and the green algal Rubisco linker protein EPYC1. NCPR, net charge per residue; Pondr, disorder prediction; hydropathy, HYDRO; PLAAC, prion-like domains.

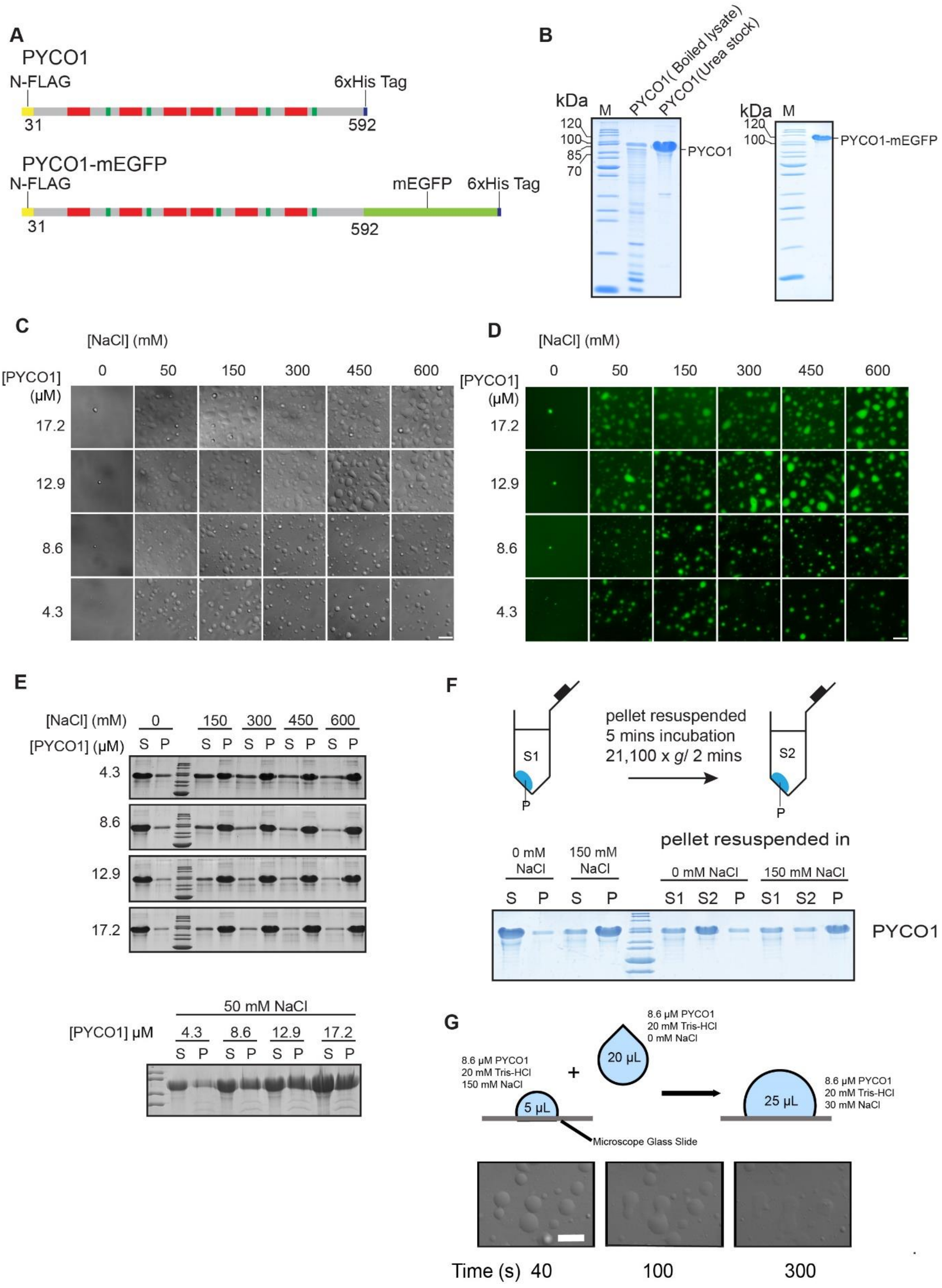

Fig. S2. Purification, Microscopy and Sedimentation of PYCO1.

(A) Scheme showing features of the recombinant PYCO1 and PYCO1-GFP proteins used. (B) (Left) Purification of PYCO1 was improved when 8 M Urea was used in the purification to circumvent degradation and purity issues. (Right) Purification of PYCO1-GFP conducted in a similar manner except no boiling was involved. (C, D) DIC (C) and GFP (D) images of PYCO1 condensates with

varying NaCl and protein concentrations. (E) Representative SDS-PAGE gels of PYCO1 sedimentation experiments using the indicated NaCl and protein concentrations in 20 mM Tris-HCl pH 8. (F) PYCO1 condensates repartition following buffer exchange into 20 mM Tris-HCl pH 8. (G) Pre-formed PYCO1 condensates dissolve upon dilution of NaCl to 30 mM. Scale bars, 15 µm.

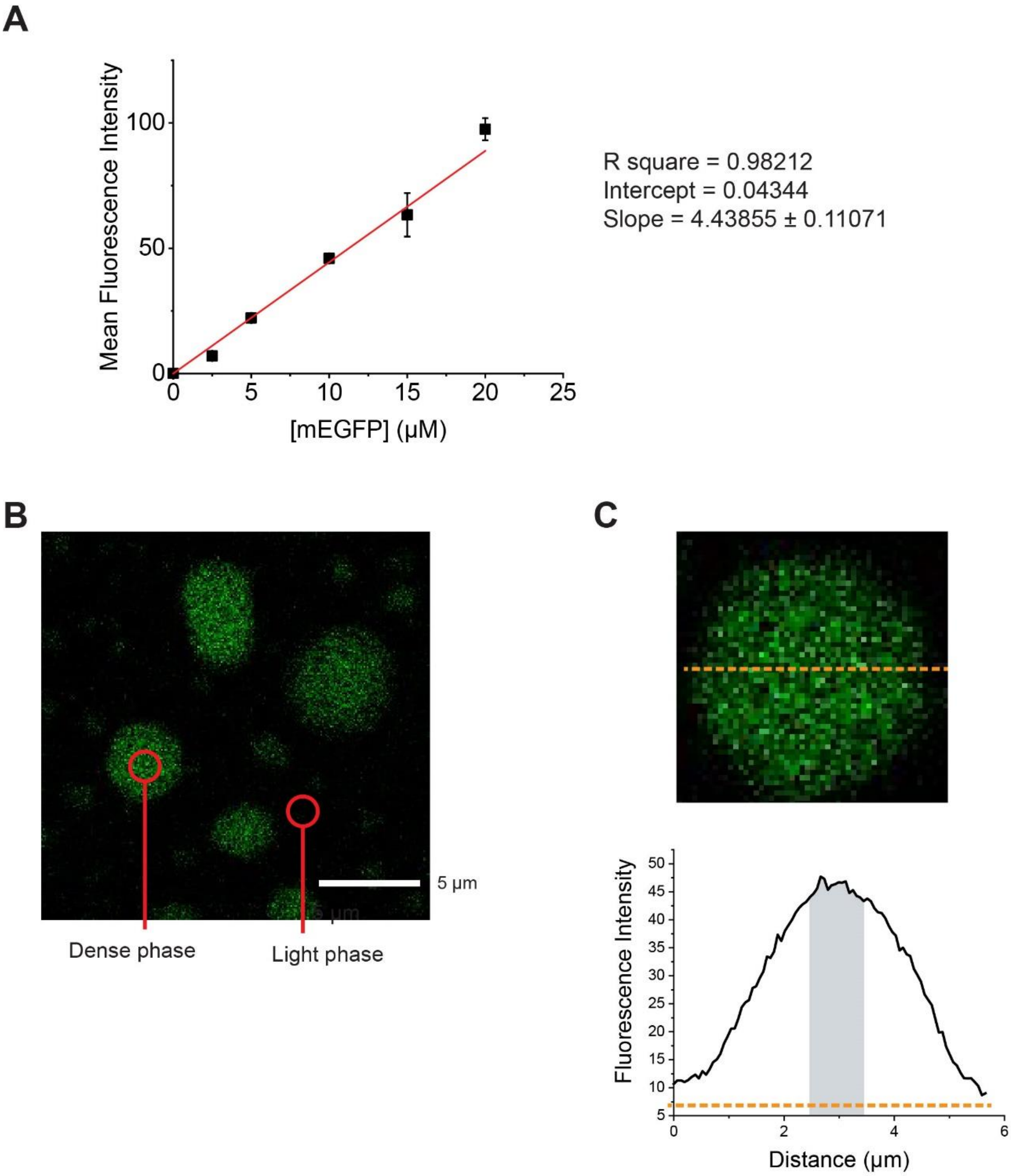

Fig. S3. Quantification of dense and light phase of the PYCO1 phase separation.

(A) Standard curve showing intensity of purified mEGFP at varying concentrations. (B) Representative image of PYCO1 condensates. Only condensates larger than 5 μm diameter were selected for analysis. (C) Representative fluorescence intensity of PYCO1 condensates. Maximum intensity was used to calculate the C_{dense} of PYCO1.

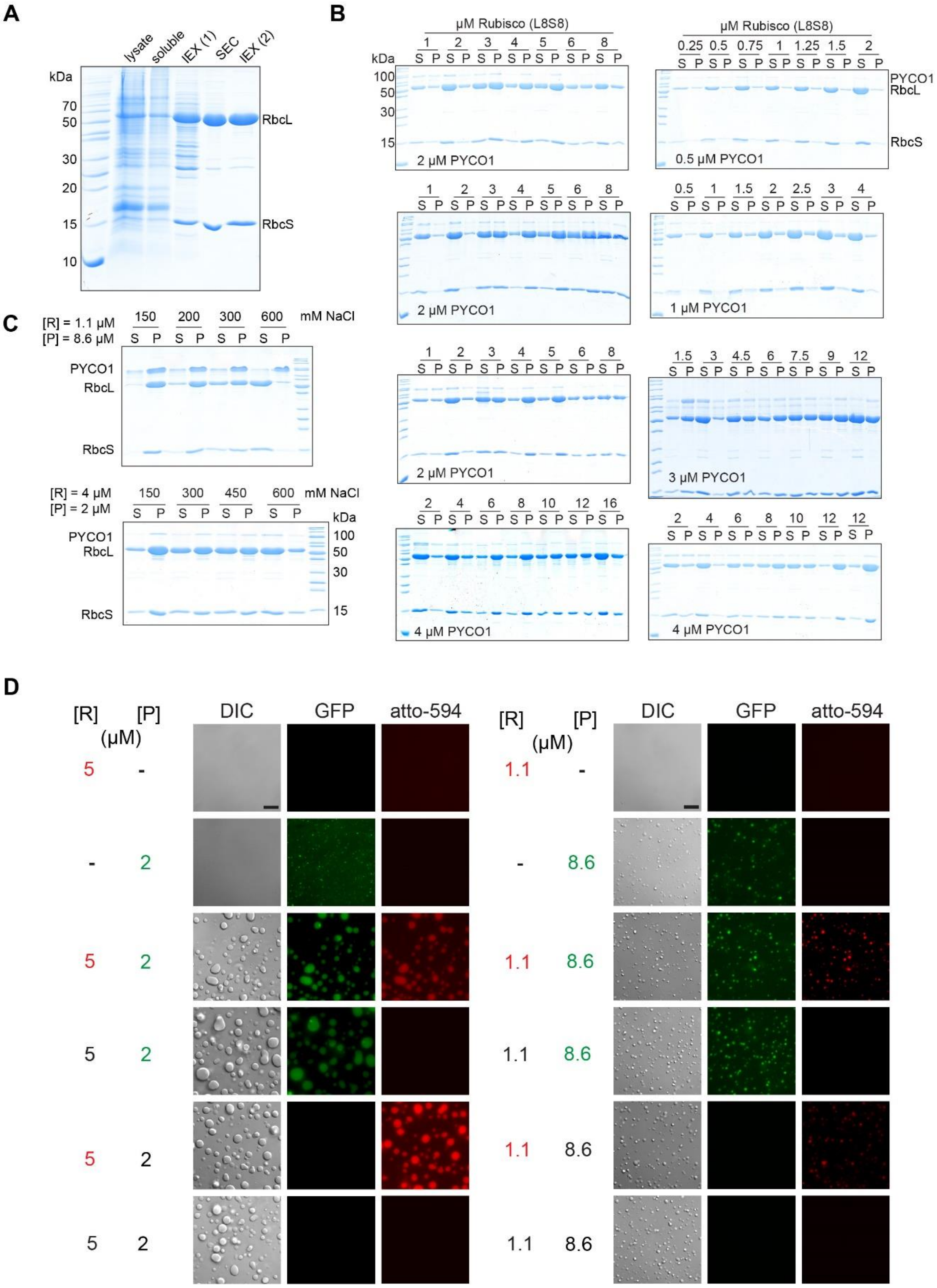

Fig. S4. Characterization of heterotypic PYCO1-Rubisco condensates. Related to Fig. 3

(A) Representative purification of *Phaeodactylum tricornutum* Rubisco from algal lysate. IEX- anion exchange chromatography; SEC- size exclusion chromatography (B) Sedimentation analysis of PYCO1-Rubisco condensates formed by using the indicated concentrations of Rubisco and PYCO1. (C) Rubisco partitioning is salt sensitive. PYCO1-Rubisco condensates were formed at the indicated

salt concentrations followed by sedimentation analysis. (D) Expanded version of Fig. 3C including additional control experiments. Fluorescent samples were labelled using less than 5% PYCO1-GFP or atto-Rubisco. Scale bar, 15 µm. [R] and [P]: respective concentration of Rubisco and PYCO1 used in the experiment.

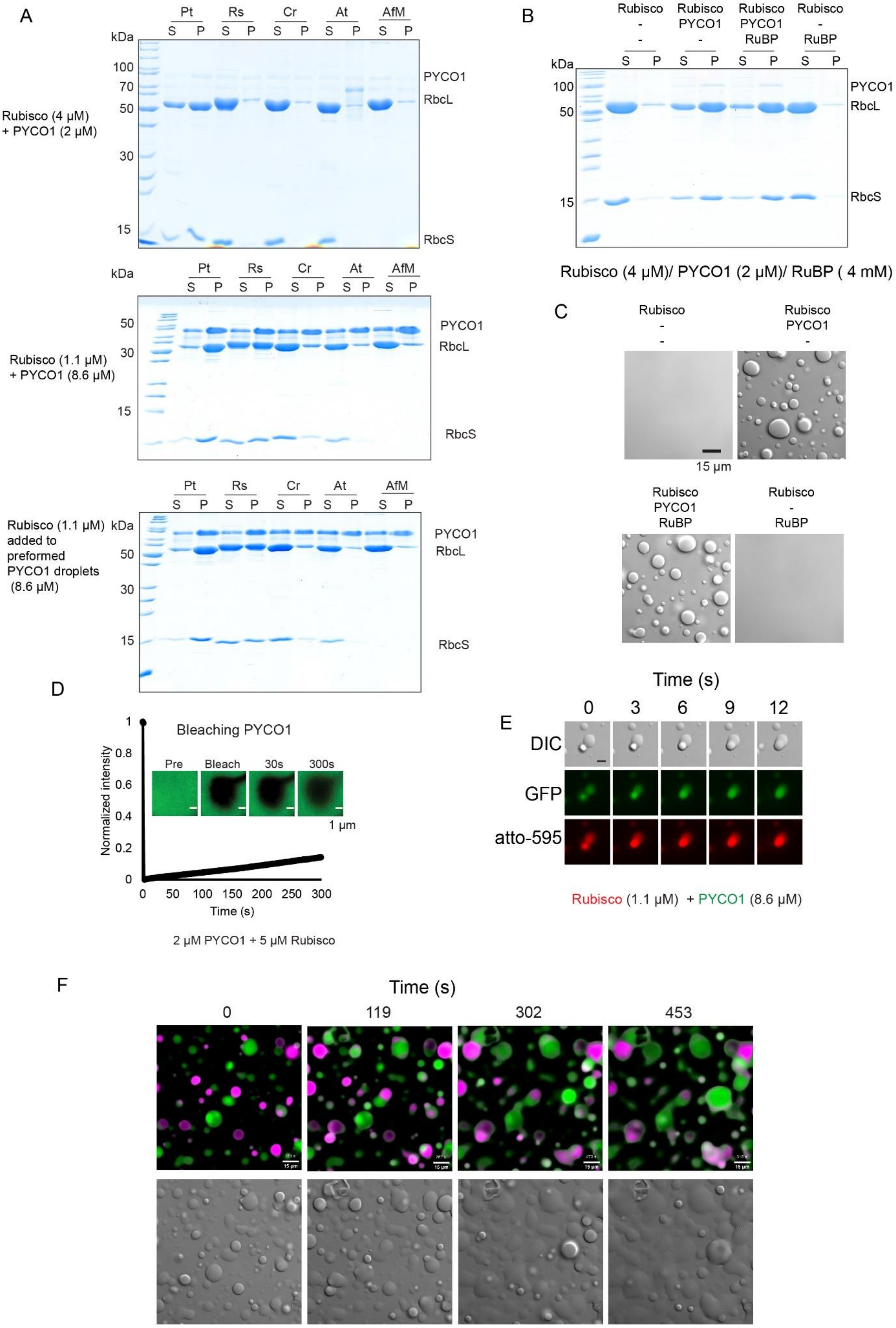

A
Pt Rs Cr At AfM
S P S P S P S P S P
kDa
100
70
50
30
15
PYCO1
RbcL
RbcS
Rubisco (4 μM)
+ PYCO1 (2 μM)
Rubisco (1.1 μM)
+ PYCO1 (8.6 μM)
Rubisco (1.1 μM) added to preformed PYCO1 droplets (8.6 μM)
B
Rubisco - -
Rubisco PYCO1 -
Rubisco PYCO1 RuBP
Rubisco - RuBP
Rubisco (4 μM)/ PYCO1 (2 μM)/ RuBP (4 mM)
C
15 μm
D
Bleaching PYCO1
Pre
Bleach
30s
300s
1 μm
Normalized intensity
Time (s)
2 μM PYCO1 + 5 μM Rubisco
E
Time (s)
0 3 6 9 12
DIC
GFP
atto-595
Rubisco (1.1 μM) + PYCO1 (8.6 μM)
F
Time (s)
0 119 302 453

Fig. S5. The specificity and activity of heterotypic condensates. Related to Fig. 3

(A) Sedimentation assays performed with various Rubiscos. Pt, *Phaeodactylum tricornutum*; Rs, *Rhodobacter sphaeroides*; Cr, *Chlamydomonas reinhardtii*; At, *Arabidopsis thaliana*; AfM, *Acidithiobacillus ferrooxidans* Form II. (B) Heterotypic condensate formation is unaffected by the presence of 4 mM RuBP and other carboxylase assay components (20 mM $NaHCO_3$, 10 mM $MgCl_2$, 110 mM NaCl, 5 mM DTT) (C) Droplet morphology is unaffected by the presence of 4 mM RuBP. (D) PYCO1 mobility is drastically reduced in condensates containing a high proportion of Rubisco. (E) Coalescence of heterotypic PYCO1-Rubisco condensates. [R]/[P] = 0.12. (F) Expanded view of experiment shown in Fig. 4D. PYCO1-mRuby and PYCO1-GFP heterotypic condensates ([R]/[P] = 2.5) were prepared separately and mixed. PYCO1-GFP and PYCO1-mRuby signals remained in distinct sectors. Scale bars, 15 µm.

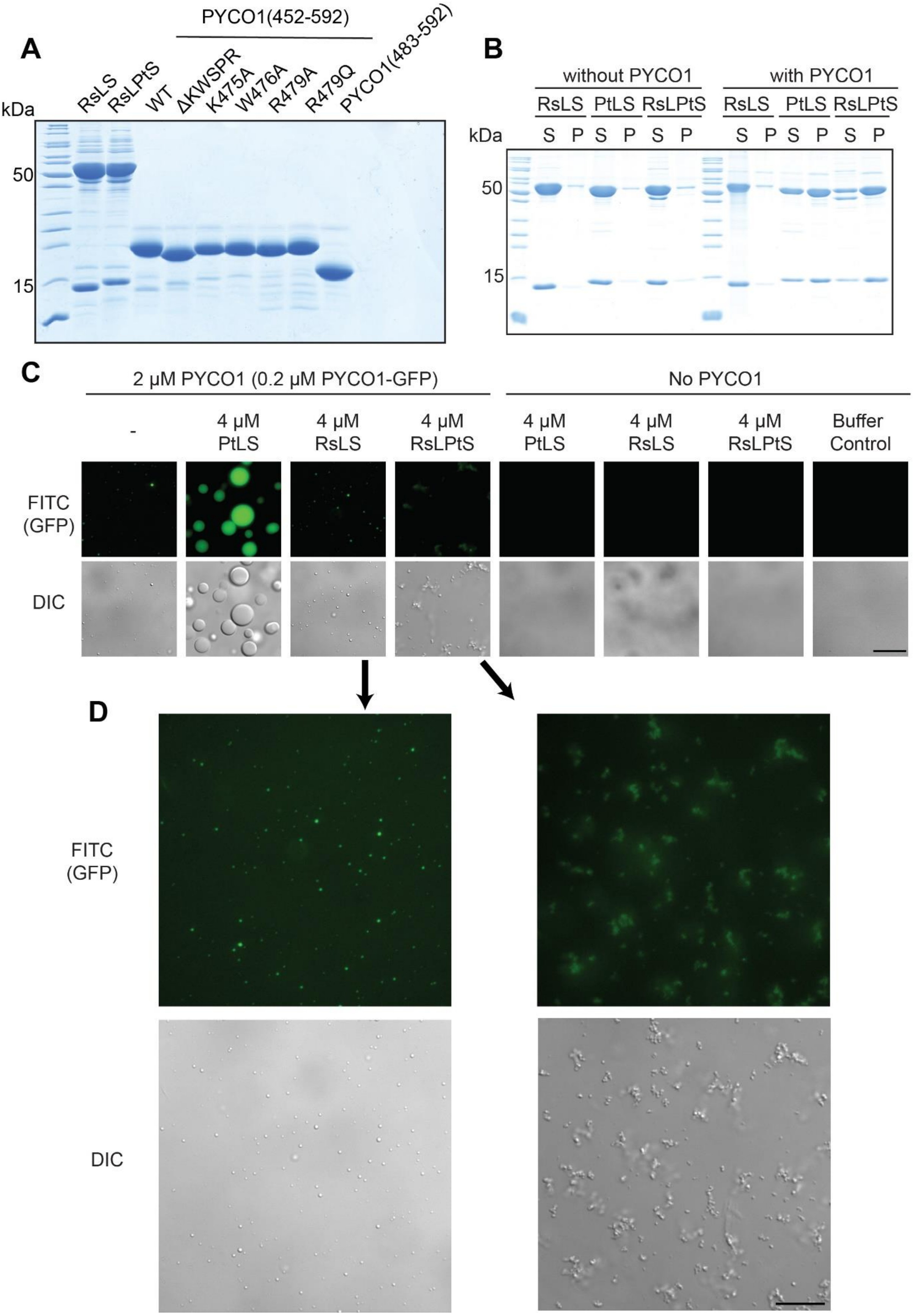

Fig. S6. Localization of PYCO1 and Rubisco stickers underlying heterotypic phase separation.

(A) SDS-PAGE analysis of purified proteins used for the analyses in Figure 5. Approximately 5 µg of each protein were loaded onto the gel. (B) Sedimentation assays of heterotypic condensate formation using different Rubiscos (4 µM) in the absence and presence of 2 µM PYCO1. (C) Microscopy analysis of heterotypic condensates. (D) Expanded field of view of the two indicated columns in (C). Scale bars, 15 µm.

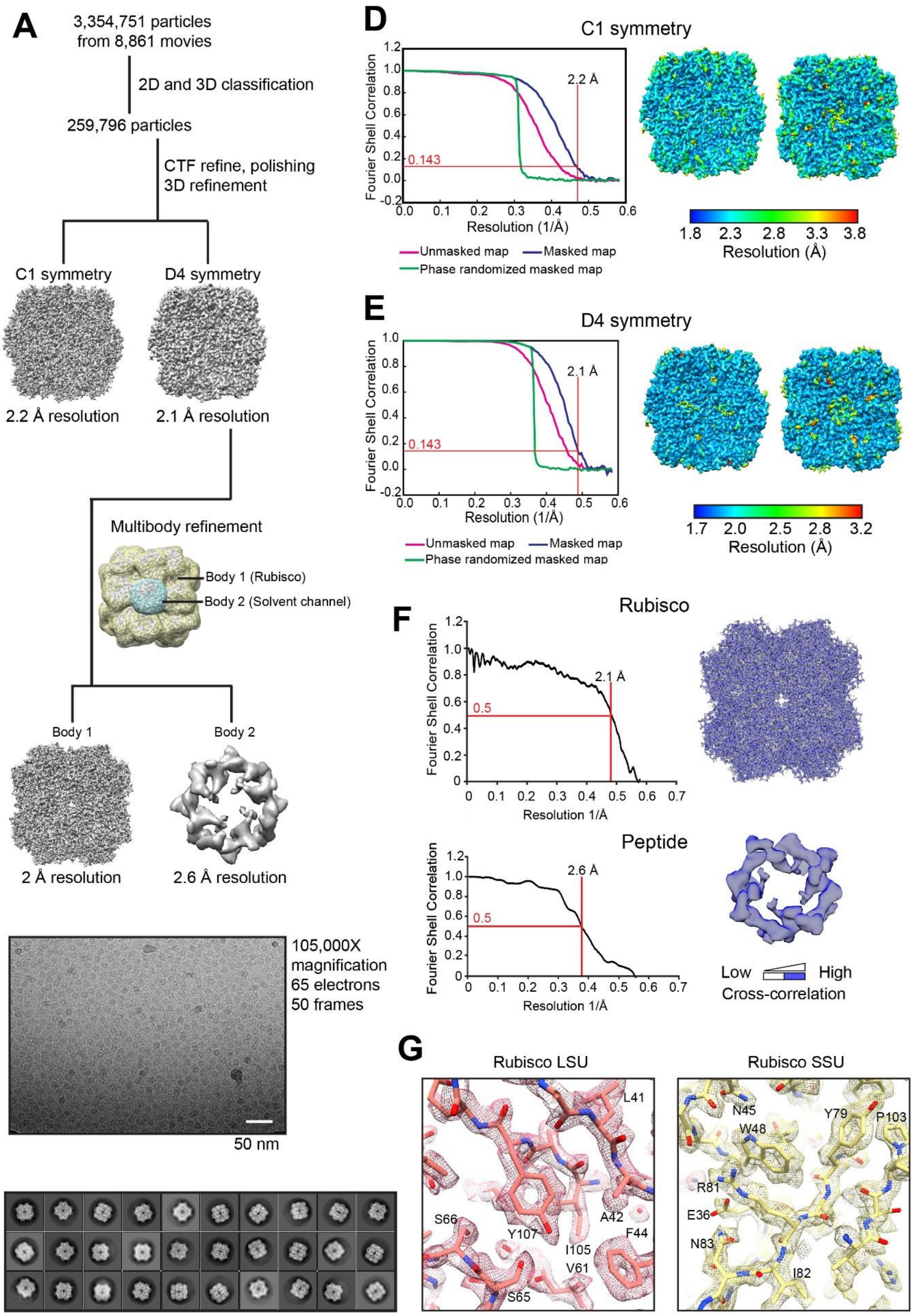
A
3,354,751 particles from 8,861 movies
2D and 3D classification
259,796 particles
CTF refine, polishing 3D refinement
C1 symmetry
D4 symmetry
2.2 Å resolution
2.1 Å resolution
Multibody refinement
Body 1 (Rubisco)
Body 2 (Solvent channel)
Body 1
Body 2
2 Å resolution
2.6 Å resolution
B
105,000X magnification
65 electrons
50 frames
50 nm
C
D
C1 symmetry
Fourier Shell Correlation
Resolution (1/Å)
2.2 Å
0.143
Unmasked map
Masked map
Phase randomized masked map
1.8 2.3 2.8 3.3 3.8
Resolution (Å)
E
D4 symmetry
2.1 Å
0.143
Unmasked map
Masked map
Phase randomized masked map
1.7 2.0 2.5 2.8 3.2
Resolution (Å)
F
Rubisco
2.1 Å
0.5
Resolution 1/Å
Peptide
2.6 Å
0.5
Low
High
Cross-correlation
G
Rubisco LSU
Rubisco SSU
L41
S66
Y107
A42
F44
I105
V61
S65
N45
W48
Y79
P103
R81
E36
N83
I82

Fig. S7. Cryo-EM Single Particle Reconstruction of the Rubisco-PYCO1(452-492) complex. Related to Fig. 5.

(A) Data-processing workflow. Particles were first subjected to 2D and 3D classification, followed with refinement and polishing. Two masks were placed on the resulting density map and multibody refined. Body 1 consisting of Rubisco had a final resolution of 2 Å, and body 2 consisting of PYCO1 fragment had a final resolution of 2.6 Å. (B) Representative micrograph of sample. 0.5 mg/mL of Rubisco incubated with 21.6 µM PYCO1(452-492) for 1 minute in 20 mM Tris pH 8.0 and 50 mM NaCl. 4 µL of the sample was placed on grids for 10 seconds, blotted and vitrified. (C) 2D classes of the selected particles. (D) FSC curve of reconstructed density map with C1 symmetry. Local resolution of the density map is shown on the right. (E) FSC curve of reconstructed density map with D4 symmetry. Local resolution of the density map is shown on the right. (F) FSC curve showing cross correlation of Rubisco (body 1) and PYCO1 peptide (body 2) after multibody refinement. (G) Density map and model of the large subunit and small subunit of Rubisco.

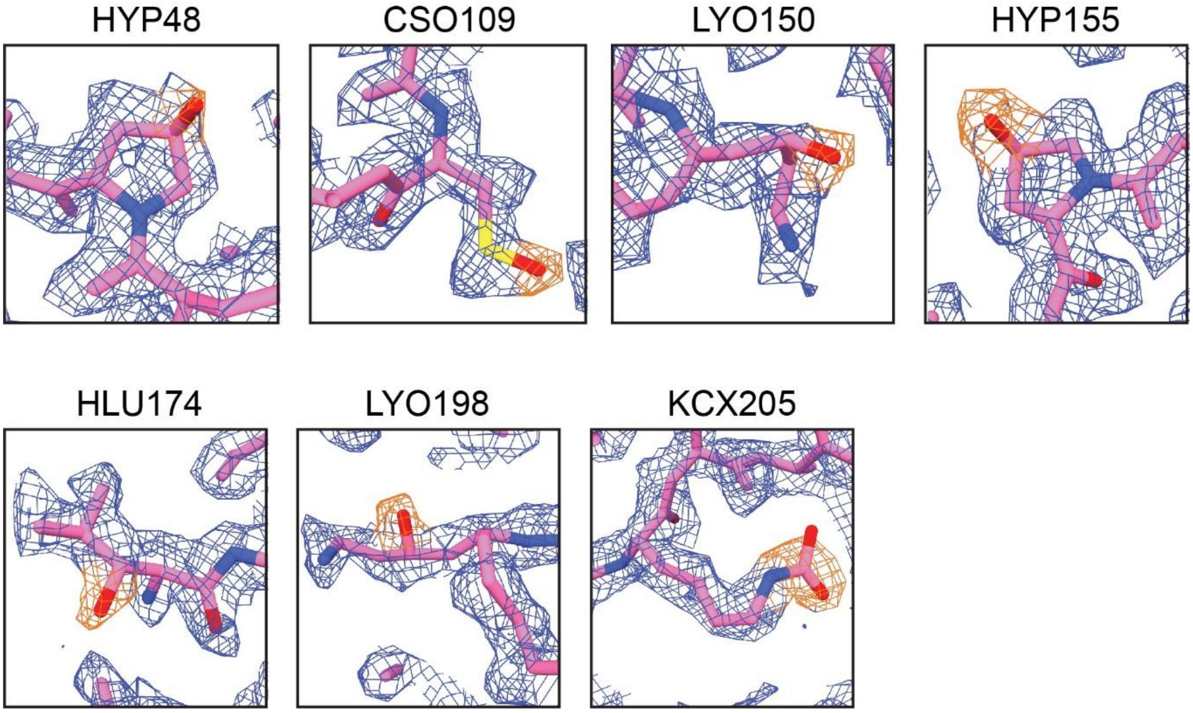

Fig. S8. ***P. tricornutum*** **Rubisco posttranslational modifications identified in the cryo-EM structure**

Density map of identified Rubisco large subunit posttranslational modifications. HYP, 4-hydroxyproline; CSO, S-hydroxycysteine; LYO, 4-hydroxylysine; HLU, β-hydroxyleucine; KCX, lysine-NZ-carboxylic acid. PTMs are shown in orange on the density map.

SUPPLEMENTAL TABLES

Table S1. Phatr3_J49957 (PYCO1) amino acid sequence variations

Source	Accession	Protein Sequence	Residue number	Changes Identified
NCBI Reference Sequence; Bowler, C. et al 2008	XP_002184702	MKISVKSATLALLMVPTTGFLHAHPARSVETAFH<br>APTHTTWKTGARWGRTPRSGSSGFSPQSAGSYA<br>GRSAFGRDSTYGSSTSVGSATAPTAPGYSSMPAK<br>VYANAGPNQKYSMTKWSPQNGASVNGGGPSAY<br>SSSNGVPAGGNGAMGTGYNPSAQSNNAYQSSAP<br>ATGSAAPTYSSMPGQAYAGSGPPKNYSMVKWS<br>PRGGATGGSIGTGYNPAQPASAASSNDAYTAPST<br>GSGAKGAPSYSSMPGQAYAGSGPRKNYSMVKW<br>SPRGGSSRASNGGGGGSPTYSSLPGQAYSGSGPG<br>KNYSMVKWSPQGGSSVSSNRGGGASAGSLGTG<br>YNPAQPTTAGGASSNESYSAPSTGGSSSGSTPSYS<br>SMPGQAHAGSGPSKNYSMVKWSPRGGSSRASN<br>GGGATGGSIGTGYNPAQAAPAASSYGSSPSSSNS<br>NAYSAPTGNAAPSYSSMPGQTNTGSGPTKRYAM<br>VKWSPRGGSVVSFNRVGGGGGSMGTGYNPAQA<br>AAPPAAAAASYSPAPPAPASSGYGNLASEWASM<br>NTEKASPAPASSAYGGGAPSPAPAPAPAPRSGNA<br>YGNLAAEWGSMNQGDSSAAF	1-583	N.A.
Sequenced PYCO1; This Study	N.A.	MKISVKSATLALLMVPTTGFLHAHPARSVETAFH<br>APTHTTWKTGARWGRTPRSGSSGFSPQSAGSYA<br>GRSAFGRDSTYGSSTSVGSATAPTAPGYSSMPAK<br>VYANAGPNQKYSMTKWSPQNGASVNGG**S**PSAY<br>SSSNGVPAGGNGA**I**GTGYNPSAQSNNAYQSSAP<br>ATGSAAPTYSSMPGQAYAGSGPPKNYSMVKWS<br>PRG**GSSRASNGG**GATGGSIGTGYNPAQPASAAS<br>SNDAYTAPSTGSGA**N**GAPSYSSMPGQAYAGSGP<br>RKNYSMVKWSPRGGSSRASNGGGGGSPTYSSLP<br>GQAYSGSGPGKNYSMVKWSPQGGSSVSSNRGG<br>GASAGSLGTGYNPAQPTTAGGASSNESYSAPSTG<br>GSSSGSTPSYSSMPGQA**Y**AGSGPSKNYSMVKWS<br>PRGGSSRASNGGGATGGSIGTGYNPAQAAPAAS<br>SYGSSPSSSNSNAYSAPTGNAAPSYSSMPGQTNT<br>GSGPTKRYAMVKWSPRGGSVVSFNRVGGGGGS<br>MGTGYNPAQAAAPPAAAAASYSPAPPAPASSGY<br>GNLASEWASMNTEKASPAPASSAYGGGAPSPAP<br>APAPAPRSGNAYGNLAAEWGSMNQGDSSAAF	1-592	G129S<br>M147I<br>Insertion of GSSRASNGG between G201 and 202<br>K246N<br>H381Y
GenBank; Bowler, C. et al 2008	CT924498	MKISVKSATLALLMVPTTGFLHAHPARSVETAFH<br>APTHTTWKTGARWGRTPRSGSSGFSPQSAGSYA<br>GRSAFGRDSTYGSSTSVGSATAPTAPGYSSMPAK<br>VYANAGPNQKYSMTKWSPQNGASVNGG**S**PSAY<br>SSSNGVPAGGNGA**I**GTGYNPSAQSNNAYQSSAP<br>ATGSAAPTYSSMPGQAYAGSGPPKNYSMVKWS<br>PRG**GSSRASNGG**GATGGSIGTGYNPAQPASAAS<br>SNDAYTAPSTGRGA**N**GAPSYSSMPGKA	1-258	G129S<br>M147I<br>Insertion of GSSRASNGG between G201 and 202<br>K246N
GenBank; Bowler, C. et al 2008	CT921751	MKISVKSATLALLMVPTTGFLHAHPARSVETAFH<br>APTHTTWKTGARWGRTPRSGSSGFSPQSAGSYA<br>GRSALGRD<br>STYGSSTNVGSATAPTAPGYSSLPAKVYANAGP<br>NQKYSMTKWSPPNGASPNGG**S**PSALHSHNRVPT<br>GSNGTLWH<br>RKQSPRPIPTTPNQNPGPPPWQCRP	1-174	G129S
	CT930415		18-232	

GenBank; Bowler, C. et al 2008		TGFLFAHPARSVETAFHAPTHTTWKTGARWGRTPRSGSSGFSPQSAGSYAGRSAFGRDSTYGSSTSVGSATAPTAP GYSSMPAKVYANAGPNQKYSMTKWSPQNGASVNGG**S**PSAYSSSNGVPAGGNGA**I**GTGYNPSAQSNNAYQSSAPATGSAAPTYSSMPGQAYAGSGPPKNYSMVKWSPRG**GSSRASNGG**GATGGSIGTGYNPAQPASAASFQRCLNGPRARVSGGQPGLPAKRSIARQALRRNRDPEERPPWVNWSPPWRHLPG		G129S M147I Insertion of GSSRASNGG between G201 and 202
GenBank; Bowler, C. et al 2008	CU703984	KGAPSYSSMPGQAYAGSGPRKNYSMVKWSPRGGSSRASNGGGGGSPTYSSLPGQAYSGSGPGKNYSMVKWSPQ GGSSVSSNRGGGASAGSLGTGYNPAQPTTAGGASSNESYSAPSTGGSSSGSTPSYSSMPGQA**Y**AGSGPSKNYSMVKWSPRGGSSRASNGGGATGGSIGTGYNPAQAAPAASSYRSSPSSSNSNAYSAPTGNAAPSYSSMPGQTTLDPVPPKPTPWSSGFPKAALSFSFNPRSAVVGRINGETEKKPGAQNGTTLVSAPPP	247-509	H381Y
GenBank; Bowler, C. et al 2008	CU705829	MKISVKSATLALLMVPTTGFLHAHPARSVETAFHAPTHTTWKTGARWGRTPRSGSSGFSPQSAGSYAGRSAFGRDSTYGSSTSVGSATAPTAPGYSSMPAKVYANAGPNQKYSMTKWSPQNGASVNGG**S**PSAYSSSNGVPAGGNGAIGTGYNPSAQSNNAYQSSAPATGSAAPTYSSMPGQAYAGSGPPKNYSMVKWSPRGGPSRALHGGRATGWSLGTGYNPAQPASAGLVPQMPTGPPARVRGANGGSHVQFSGPEKTLARKGTSAKKTPMGKGGSPPGRGPPPGPSPGGGGGKTSPLLHKFSPSPGGEGFISRGGSRAPPAGQKKNHYSSQRGVVQ	1-330	G129S
GenBank; Bowler, C. et al 2008	CU706648	GAMGTGYNPSAQSNNAYQSSAPATGSAAPTYSSMPGQAYAGSGPPKNYSMVKWSPRGGATGGSIGTGYNPAQPASAASSNDAYTAPSTGSGAKGAPSYSSMPGQAYAGSGPRKNYSMVKWSPRGGSSRASNGGGGGSPTYSSLPGQAYSGSGPGKNYSMVKWSPQGGSSVSSNRGGGASAGSLGTGYNPAQPTTAGGASSNESYSAPSTGGSSSGSTPSYSSMPGQA**Y**AGIGTFQELLHGQVVAPWGLLPGLQRWAGPWGVPSAPGYNRARQHPRPRPTDPPFPVKSNANRHPPAMAPQLTVPAREKANGNRVHQGPTPCSMGPPKSGPCGLLYTRSAGLGKTLGKPE	145-492	H381Y

Table S2. PYCO1 dense phase and light phase quantification

**Condensate type**	**Dense phase mean max fluorescence intensity**	**Light phase mean intensity**	**C_{dense}**	**C_{light}**	**Partition Coefficient**
Homotypic (n=90)	53 ± 1.5	0.6 ± 0.15	575 ± 160 µM	5.7 ± 1.5 µM	100
Heterotypic [R]/[P] = 0.06 (n=30)	30 ± 1.5	0.2 ± 0.1	350 ± 20 µM	1.8 ± 0.5 µM	200
Heterotypic [R]/[P] = 1.5 (n=30)	10 ± 0.4	0.12 ± 0.02	30 ± 1 µM	0.24 ± 0.05 µM	125
Heterotypic [R]/[P] = 2.5 (n=30)	8 ± 0.15	0.17 ± 0.01	21 ± 0.4 µM	0.37 ± 0.04 µM	57

Errors are given as SEM

Table S3. Rubisco dense phase and light phase quantification

**Condensate type**	**Fraction of Rubisco in pellet**	**Mass of Rubisco in pellet**	**Mass of PYCO1 in pellet**	**PYCO1 C_{dense}**	**Volume fraction**	**Rubisco C_{dense}**	**Rubisco C_{light}**	**Partition Coefficient**
Heterotypic [R]/[P] = 0.06	0.9 ± 0.05	1.3 ± 0.1 µg	2 µg	20 µg/µL	0.1 µL	13 ± 0.7 µg/µL; 24 ± 1.2 µM	0.04 µg/µL; 0.07 µM	330
Heterotypic [R]/[P] = 1.5	0.5 ± 0.05	4.3 ± 0.4 µg	0.3 µg	1.6 µg/µL	0.2 µL	25 ± 2.6 µg/µL; 45 ± 4.6 µM	0.84 µg/µL; 1.5 µM	30
Heterotypic [R]/[P] = 2.5	0.7 ± 0.05	9.3 ± 0.7 µg	0.4 µg	1.3 µg/µL	0.3 µL	32 ± 2.4 µg/µL; 58 ± 4.3 µM	1.0 µg/µL; 1.8 µM	32

Errors are S.D., n=3.

Table S4. Cryo-EM Statistics and Model Validation

Data collection and processing	Rubisco	PYCO1(452-592)
Magnification	105,000X	105,000X
Voltage (kV)	300	300
Defocus range (μm)	-0.8 to -1.6	-0.8 to -1.6
Pixel size (Å)	0.858	0.858
Total extracted particles (no.)	3,354,751	3,354,751
Total movies (no.)	8,861	8,861
Refined particles (no.)	259,796	259,796
Final particles (no.)	259,796	259,796
Symmetry imposed	D4	D4
Map sharpening *B* factor ($Å^2$)	80	65
Map resolution (Å)<br><br>FSC threshold	2Å<br><br>0.143	2.7 Å<br><br>0.143
**Model Refinement**		
Model resolution cut-off (Å)	Same as focused maps	Same as focused maps
Model composition<br>Nonhydrogen atoms<br>Protein residues<br>Ligands	 39072<br>4960<br>CAP=8	 244
R.M.S. deviations<br>Bond Lengths (Å)<br>Bond angles (°)	 0.004<br>0.730	 0.074<br>8.430
Validation<br>Molprobity score<br>Clashscore<br>Poor rotamers (%)<br>CαBLAM outliers (%)	 0.80<br>0.43<br>1.18<br>1.47	 3.07<br>8.4<br>16.67<br>0
Ramachandran plot<br>Favored (%)<br>Allowed (%)<br>Disallowed (%)	 97.55<br>2.45<br>0.00	 83.33<br>16.67<br>0.00

Table S5. Primers used in this study. See Table S5.xls

Table S6. Plasmids used in this study and selected protein sequences

Plasmid	Protein	Encoded Protein Sequence
pPtPuc3_*FcpB*<br>*_PYCO1ECFP*<br>*_FcpA*	PYCO1-<br>ECFP	MKISVKSATLALLMVPTTGFLHAHPARSVETAFHAPTHTTW<br>KTGARWGRTPRSGSSGFSPQSAGSYAGRSAFGRDSTYGSSTS<br>VGSATAPTAPGYSSMPAKVYANAGPNQKYSMTKWSPQNGA<br>SVNGGSPSAYSSSNGVPAGGNGAIGTGYNPSAQSNNAYQSSA<br>PATGSAAPTYSSMPGQAYAGSGPPKNYSMVKWSPRGGSSRA<br>SNGGGATGGSIGTGYNPAQPASAASSNDAYTAPSTGSGANG<br>APSYSSMPGQAYAGSGPRKNYSMVKWSPRGGSSRASNGGG<br>GGSPTYSSLPGQAYSGSGPGKNYSMVKWSPQGGSSVSSNRG<br>GGASAGSLGTGYNPAQPTTAGGASSNESYSAPSTGGSSSGST<br>PSYSSMPGQAYAGSGPSKNYSMVKWSPRGGSSRASNGGGAT<br>GGSIGTGYNPAQAAPAASSYGSSPSSSNSNAYSAPTGNAAPS<br>YSSMPGQTNTGSGPTKRYAMVKWSPRGGSVVSFNRVGGGG<br>GSMGTGYNPAQAAAPPAAAAASYSPAPPAPASSGYGNLASE<br>WASMNTEKASPAPASSAYGGGAPSPAPAPAPAPRSGNAYGN<br>LAAEWGSMNQGDSSAAFWRADPAFLYKVVISNSCRSTLEDP<br>RVPVATMVSKGEELFTGVVPILVELDGDVNGHKFSVSGEGE<br>GDATYGKLTLKFICTTGKLPVPWPTLVTTLTWGVQCFSRYPD<br>HMKQHDFFKSAMPEGYVQERTIFFKDDGNYKTRAEVKFEGD<br>TLVNRIELKGIDFKEDGNILGHKLEYNYISHNVYITADKQKNG<br>IKANFKIRHNIEDGSVQLADHYQQNTPIGDGPVLLPDNHYLST<br>QSALSKDPNEKRDHMVLLEFVTAAGITLGMDELYK*
pET24b*FLAG*<br>*PYCO1ΔN30H*<br>*is6*	PYCO1	MASM*DYKDDDDK*AGRGGSEFTAFHAPTHTTWKTGARWGRT<br>PRSGSSGFSPQSAGSYAGRSAFGRDSTYGSSTSVGSATAPTAP<br>GYSSMPAKVYANAGPNQKYSMTKWSPQNGASVNGGSPSAY<br>SSSNGVPAGGNGAIGTGYNPSAQSNNAYQSSAPATGSAAPTY<br>SSMPGQAYAGSGPPKNYSMVKWSPRGGSSRASNGGGATGG<br>SIGTGYNPAQPASAASSNDAYTAPSTGSGANGAPSYSSMPGQ<br>AYAGSGPRKNYSMVKWSPRGGSSRASNGGGGGSPTYSSLPG<br>QAYSGSGPGKNYSMVKWSPQGGSSVSSNRGGGASAGSLGTG<br>YNPAQPTTAGGASSNESYSAPSTGGSSSGSTPSYSSMPGQAY<br>AGSGPSKNYSMVKWSPRGGSSRASNGGGATGGSIGTGYNPA<br>QAAPAASSYGSSPSSSNSNAYSAPTGNAAPSYSSMPGQTNTG<br>SGPTKRYAMVKWSPRGGSVVSFNRVGGGGGSMGTGYNPAQ<br>AAAPPAAAAASYSPAPPAPASSGYGNLASEWASMNTEKASP<br>APASSAYGGGAPSPAPAPAPAPRSGNAYGNLAAEWGSMNQG<br>DSSAAFKLAAALE*HHHHHH**
pET24b*FLAG*<br>*PYCO1ΔN30m*<br>*EGFPHis6*	PYCO1-<br>GFP	MASM*DYKDDDDK*AGRGGSEFTAFHAPTHTTWKTGARWGRT<br>PRSGSSGFSPQSAGSYAGRSAFGRDSTYGSSTSVGSATAPTAP<br>GYSSMPAKVYANAGPNQKYSMTKWSPQNGASVNGGSPSAY<br>SSSNGVPAGGNGAIGTGYNPSAQSNNAYQSSAPATGSAAPTY<br>SSMPGQAYAGSGPPKNYSMVKWSPRGGSSRASNGGGATGG<br>SIGTGYNPAQPASAASSNDAYTAPSTGSGANGAPSYSSMPGQ<br>AYAGSGPRKNYSMVKWSPRGGSSRASNGGGGGSPTYSSLPG<br>QAYSGSGPGKNYSMVKWSPQGGSSVSSNRGGGASAGSLGTG<br>YNPAQPTTAGGASSNESYSAPSTGGSSSGSTPSYSSMPGQAY<br>AGSGPSKNYSMVKWSPRGGSSRASNGGGATGGSIGTGYNPA<br>QAAPAASSYGSSPSSSNSNAYSAPTGNAAPSYSSMPGQTNTG<br>SGPTKRYAMVKWSPRGGSVVSFNRVGGGGGSMGTGYNPAQ

		AAAPPAAAAASYSPAPPAPASSGYGNLASEWASMNTEKASP<br>APASSAYGGGAPSPAPAPAPAPRSGNAYGNLAAEWGSMNQG<br>DSSAAFKLGSLGGSGGRMVSKGEELFTGVVPILVELDGDVNG<br>HKFSVRGEGEGDATNGKLTLKFICTTGKLPVPWPTLVTTLTY<br>GVQCFSRYPDHMKQHDFFKSAMPEGYVQERTISFKDDGTYK<br>TRAEVKFEGDTLVNRIELKGIDFKEDGNILGHKLEYNFNSHN<br>VYITADKQKNGIKANFKIRHNVEDGSVQLADHYQQNTPIGDG<br>PVLLPDNHYLSTQSKLSKDPNEKRDHMVLLEFVTAAGITLGM<br>DELYKAAALE_HHHHHH_*
pET24b_FLAG PYCO1ΔN30mRubyHis6_	PYCO1-mRuby	MASM_DYKDDDDK_AGRGGSEFTAFHAPTHTTWKTGARWGRT<br>PRSGSSGFSPQSAGSYAGRSAFGRDSTYGSSTSVGSATAPTAP<br>GYSSMPAKVYANAGPNQKYSMTKWSPQNGASVNGGSPSAY<br>SSSNGVPAGGNGAIGTGYNPSAQSNNAYQSSAPATGSAAPTY<br>SSMPGQAYAGSGPPKNYSMVKWSPRGGSSRASNGGGATGG<br>SIGTGYNPAQPASAASSNDAYTAPSTGSGANGAPSYSSMPGQ<br>AYAGSGPRKNYSMVKWSPRGGSSRASNGGGGGSPTYSSLPG<br>QAYSGSGPGKNYSMVKWSPQGGSSVSSNRGGGASAGSLGTG<br>YNPAQPTTAGGASSNESYSAPSTGGSSSGSTPSYSSMPGQAY<br>AGSGPSKNYSMVKWSPRGGSSRASNGGGATGGSIGTGYNPA<br>QAAPAASSYGSSPSSSNSNAYSAPTGNAAPSYSSMPGQTNTG<br>SGPTKRYAMVKWSPRGGSVVSFNRVGGGGGSMGTGYNPAQ<br>AAAPPAAAAASYSPAPPAPASSGYGNLASEWASMNTEKASP<br>APASSAYGGGAPSPAPAPAPAPRSGNAYGNLAAEWGSMNQG<br>DSSAAFKLGSLGGSGGRMVSKGEELIKENMRMKVVMEGSV<br>NGHQFKCTGEGEGRPYEGVQTMRIKVIEGGPLPFAFDILATSF<br>MYGSRTFIKYPADIPDFFKQSFPEGFTWERVTRYEDGGVVTV<br>TQDTSLEDGELVYNVKVRGVNFPSNGPVMQKKTKGWEPNT<br>EMMYPADGGLRGYTDIALKVDGGGHLHCNFVTTYRSKKTV<br>GNIKMPGVHAVDHRLERIEESDNETYVVQREVAVAKYSNLG<br>GGMDELYKAAALE_HHHHHH_*
pET24b_FLAG PYCO1(452-592)His6_	PYCO1(452-592)	MASM_DYKDDDDK_AGRGGSEFPSYSSMPGQTNTGSGPTKRYA<br>MVKWSPRGGSVVSFNRVGGGGGSMGTGYNPAQAAAPPAAA<br>AASYSPAPPAPASSGYGNLASEWASMNTEKASPAPASSAYG<br>GGAPSPAPAPAPAPRSGNAYGNLAAEWGSMNQGDSSAAFKL<br>AAALE_HHHHHH_*
pET24b_FLAG PYCO1(483-592)His6_	PYCO1(483-592)	MASM_DYKDDDDK_AGRGGSEFVVSFNRVGGGGGSMGTGYNP<br>AQAAAPPAAAAASYSPAPPAPASSGYGNLASEWASMNTEKA<br>SPAPASSAYGGGAPSPAPAPAPAPRSGNAYGNLAAEWGSMN<br>QGDSSAAFKLAAALE_HHHHHH_*
pET24b_PYCO1ΔN30His6_	PYCO1 WT	MASMAGRGGSEFTAFHAPTHTTWKTGARWGRTPRSGSSGFS<br>PQSAGSYAGRSAFGRDSTYGSSTSVGSATAPTAPGYSSMPAK<br>VYANAGPNQKYSMTKWSPQNGASVNGGSPSAYSSSNGVPA<br>GGNGAIGTGYNPSAQSNNAYQSSAPATGSAAPTYSSMPGQA<br>YAGSGPPKNYSMVKWSPRGGSSRASNGGGATGGSIGTGYNP<br>AQPASAASSNDAYTAPSTGSGANGAPSYSSMPGQAYAGSGP<br>RKNYSMVKWSPRGGSSRASNGGGGGSPTYSSLPGQAYSGSG<br>PGKNYSMVKWSPQGGSSVSSNRGGGASAGSLGTGYNPAQPT<br>TAGGASSNESYSAPSTGGSSSGSTPSYSSMPGQAYAGSGPSK<br>NYSMVKWSPRGGSSRASNGGGATGGSIGTGYNPAQAAPAAS<br>SYGSSPSSSNSNAYSAPTGNAAPSYSSMPGQTNTGSGPTKRY<br>AMVKWSPRGGSVVSFNRVGGGGGSMGTGYNPAQAAAPPAA<br>AAASYSPAPPAPASSGYGNLASEWASMNTEKASPAPASSAY

		GGGAPSPAPAPAPAPRSGNAYGNLAAEWGSMNQGDSSAAFK<br>LAAALE*HHHHHH**
pHue*mEGFP*	*mEGFP*	SEFELGTVDGSLGGSGGRMVSKGEELFTGVVPILVELDGDVN<br>GHKFSVRGEGEGDATNGKLTLKFICTTGKLPVPWPTLVTTLT<br>YGVQCFSRYPDHMKQHDFFKSAMPEGYVQERTISFKDDGTY<br>KTRAEVKFEGDTLVNRIELKGIDFKEDGNILGHKLEYNFNSH<br>NVYITADKQKNGIKANFKIRHNVEDGSVQLADHYQQNTPIGD<br>GPVLLPDNHYLSTQSKLSKDPNEKRDHMVLLEFVTAAGITLG<br>MDELYK*
pTrc*RsLS*	*Rs*Rubisc<br>o	RsL:<br>MDTKTTEIKGKERYKAGVLKYAQMGYWDGDYVPKDTDVL<br>ALFRITPQEGVDPVEAAAAVAGESSTATWTVVWTDRLTACD<br>SYRAKAYRVEPVPGTPGQYFCYVAYDLILFEEGSIANLTASII<br>GNVFSFKPLKAARLEDMRFPVAYVKTYKGPPTGIVGERERLD<br>KFGKPLLGATTKPKLGLSGKNYGRVVYEGLKGGLDFMKDD<br>ENINSQPFMHWRDRFLYVMEAVNLASAQTGEVKGHYLNITA<br>GTMEEMYRRAEFAKSLGSVIVMVDLIIGYTAIQSISEWCRQN<br>DMILHMHRAGHGTYTRQKNHGISFRVIAKWLRLAGVDHLHC<br>GTAVGKLEGDPLTVQGYYNVCREPFNTVDLPRGIFFEQDWA<br>DLRKVMPVASGGIHAGQMHQLLSLFGDDVVLQFGGGTIGHP<br>MGIQAGATANRVALEAMVLARNEGRNIDVEGPEILRAAAKW<br>CKPLEAALDTWGNITFNYTSTDTSDFVPTASVAM*<br>RsS:<br>MRITQGCFSFLPDLTDAQISAQVDYCLGRGWAVSLEHTDDPH<br>PRNTYWEMWGMPMFDLRDPKGVMIELDECRKAWPGRYIRI<br>NAFDSTRGFETVTMSFIVNRPEVEPSLRMERTEVDGRSIRYTH<br>SIVR*
pTrc*RsLPtS*	*Rs*L*Pt*S	RsL:<br>MDTKTTEIKGKERYKAGVLKYAQMGYWDGDYVPKDTDVL<br>ALFRITPQEGVDPVEAAAAVAGESSTATWTVVWTDRLTACD<br>SYRAKAYRVEPVPGTPGQYFCYVAYDLILFEEGSIANLTASII<br>GNVFSFKPLKAARLEDMRFPVAYVKTYKGPPTGIVGERERLD<br>KFGKPLLGATTKPKLGLSGKNYGRVVYEGLKGGLDFMKDD<br>ENINSQPFMHWRDRFLYVMEAVNLASAQTGEVKGHYLNITA<br>GTMEEMYRRAEFAKSLGSVIVMVDLIIGYTAIQSISEWCRQN<br>DMILHMHRAGHGTYTRQKNHGISFRVIAKWLRLAGVDHLHC<br>GTAVGKLEGDPLTVQGYYNVCREPFNTVDLPRGIFFEQDWA<br>DLRKVMPVASGGIHAGQMHQLLSLFGDDVVLQFGGGTIGHP<br>MGIQAGATANRVALEAMVLARNEGRNIDVEGPEILRAAAKW<br>CKPLEAALDTWGNITFNYTSTDTSDFVPTASVAM*<br>PtS:<br>MRLTQGCFSFLPDLTDQQIEKQIAYCITKGWAMNVEWTDDP<br>HPRNSYWELWGLPLFDVKDPASVMFELREARKSCAAGYIRI<br>NAFNAAYGTESCVMSFIVNRPSNEPGFYLERQELEGRRIAYTT<br>KSYSVQANPEGGRY*

Sequence of FLAG and His_6 tags are in italics, linker sequences between protein of interest and fluorescent proteins as well as redundant sequences are underlined.

Movie S1. FRAP of diatom cells expressing PYCO1-CFP.

Photobleaching of a *P. tricornutum* cell carrying pPtPuc3*FcpBPYCO1ECFPFcpA*. Fluorescently tagged PYCO1 was imaged and bleached at 458 nm and 405 nm. Bleached cells were allowed to recover for 25 seconds. ECFP channel is shown first, followed by the brightfield channel.

Movie S2. Coalescence of PYCO1 homotypic condensates.

8.6 µM of PYCO1 (5% PYCO1-mEGFP) was prepared in 20 mM Tris-HCl pH 8.0 and 150 mM NaCl in 5 µL volume. Condensates were imaged using DIC and GFP channels and merged.

Movie S3. Coalescence of PYCO1-Rubisco heterotypic condensates.

2 µM of PYCO1 (5% PYCO1-mEGFP, or 5% PYCO1-mRuby) and 5 µM of Rubisco was prepared in 20 mM Tris-HCl pH 8.0 and 150 mM NaCl in 5 µL volume. Condensates were imaged using GFP and mCherry channels and subsequently merged. Two video sets are shown consecutively.

SI References

1. C. Bowler *et al.*, The Phaeodactylum genome reveals the evolutionary history of diatom genomes. *Nature* (2008).
2. R. R. Guillard, J. H. Ryther, Studies of marine planktonic diatoms. I. Cyclotella nana Hustedt, and Detonula confervacea (cleve) Gran. *Canadian journal of microbiology* **8**, 229-239 (1962).
3. R. R. L. Guillard, "Culture of Phytoplankton for Feeding Marine Invertebrates" in Culture of Marine Invertebrate Animals: Proceedings — 1st Conference on Culture of Marine Invertebrate Animals Greenport, W. L. Smith, M. H. Chanley, Eds. (Springer US, Boston, MA, 1975), 10.1007/978-1-4615-8714-9_3, pp. 29-60.
4. K. Edwards, C. Johnstone, C. Thompson, A simple and rapid method for the preparation of plant genomic DNA for PCR analysis. *Nucleic Acids Res* **19**, 1349-1349 (1991).
5. Y.-C. C. Tsai, M. C. Lapina, S. Bhushan, O. Mueller-Cajar, Identification and characterization of multiple rubisco activases in chemoautotrophic bacteria. *Nature Communications* **6**, 8883 (2015).
6. M. Siaut *et al.*, Molecular toolbox for studying diatom biology in Phaeodactylum tricornutum. *Gene* **406**, 23-35 (2007).
7. B. J. Karas *et al.*, Designer diatom episomes delivered by bacterial conjugation. *Nature communications* **6**, 6925 (2015).
8. O. Mueller-Cajar *et al.*, Structure and function of the AAA+ protein CbbX, a red-type Rubisco activase. *Nature* **479**, 194-199 (2011).
9. T. Wunder, S. L. H. Cheng, S. K. Lai, H. Y. Li, O. Mueller-Cajar, The phase separation underlying the pyrenoid-based microalgal Rubisco supercharger. *Nat Commun* **9**, 5076 (2018).
10. A.-M. Catanzariti, T. A. Soboleva, D. A. Jans, P. G. Board, R. T. Baker, An efficient system for high-level expression and easy purification of authentic recombinant proteins. *Protein Sci* **13**, 1331-1339 (2004).
11. N. Atkinson *et al.*, The pyrenoidal linker protein EPYC1 phase separates with hybrid Arabidopsis-Chlamydomonas Rubisco through interactions with the algal Rubisco small subunit. *J Exp Bot* 10.1093/jxb/erz275 (2019).
12. R. E. Diner, V. A. Bielinski, C. L. Dupont, A. E. Allen, P. D. Weyman, Refinement of the Diatom Episome Maintenance Sequence and Improvement of Conjugation-Based DNA Delivery Methods. *Front Bioeng Biotechnol* **4**, 65 (2016).
13. A. Shevchenko, H. Tomas, J. Havlis, J. V. Olsen, M. Mann, In-gel digestion for mass spectrometric characterization of proteins and proteomes. *Nat Protoc* **1**, 2856-2860 (2006).
14. T. N. Petersen, S. Brunak, G. von Heijne, H. Nielsen, SignalP 4.0: discriminating signal peptides from transmembrane regions. *Nature Methods* **8**, 785-786 (2011).
15. A. Gruber, G. Rocap, P. G. Kroth, E. V. Armbrust, T. Mock, Plastid proteome prediction for diatoms and other algae with secondary plastids of the red lineage. *The Plant Journal* **81**, 519-528 (2015).
16. A. Rastogi *et al.*, Integrative analysis of large scale transcriptome data draws a comprehensive landscape of Phaeodactylum tricornutum genome and evolutionary origin of diatoms. *Scientific Reports* **8**, 4834 (2018).
17. A. S. Holehouse, R. K. Das, J. N. Ahad, M. O. Richardson, R. V. Pappu, CIDER: Resources to Analyze Sequence-Ensemble Relationships of Intrinsically Disordered Proteins. *Biophys J* **112**, 16-21 (2017).
18. P. Romero *et al.*, Sequence complexity of disordered protein. *Proteins* **42**, 38-48 (2001).
19. J. Kyte, R. F. Doolittle, A simple method for displaying the hydropathic character of a protein. *J Mol Biol* **157**, 105-132 (1982).
20. A. K. Lancaster, A. Nutter-Upham, S. Lindquist, O. D. King, PLAAC: a web and command-line application to identify proteins with prion-like amino acid composition. *Bioinformatics* **30**, 2501-2502 (2014).

21. J. Schindelin *et al.*, Fiji: an open-source platform for biological-image analysis. *Nature Methods* **9**, 676-682 (2012).
22. J. Zivanov *et al.*, New tools for automated high-resolution cryo-EM structure determination in RELION-3. *Elife* **7** (2018).
23. S. Q. Zheng *et al.*, MotionCor2: anisotropic correction of beam-induced motion for improved cryo-electron microscopy. *Nature methods* **14**, 331-332 (2017).
24. A. Rohou, N. Grigorieff, CTFFIND4: Fast and accurate defocus estimation from electron micrographs. *Journal of structural biology* **192**, 216-221 (2015).
25. K. Valegård *et al.*, Structural and functional analyses of Rubisco from arctic diatom species reveal unusual posttranslational modifications. *The Journal of biological chemistry* **293**, 13033-13043 (2018).
26. N. Stein, CHAINSAW: a program for mutating pdb files used as templates in molecular replacement. *Journal of Applied Crystallography* **41**, 641-643 (2008).
27. P. D. Adams *et al.*, PHENIX: a comprehensive Python-based system for macromolecular structure solution. *Acta crystallographica. Section D, Biological crystallography* **66**, 213-221 (2010).
28. P. Emsley, B. Lohkamp, W. G. Scott, K. Cowtan, Features and development of Coot. *Acta crystallographica. Section D, Biological crystallography* **66**, 486-501 (2010).
29. J. Jumper *et al.*, Highly accurate protein structure prediction with AlphaFold. *Nature* **596**, 583-589 (2021).
30. E. F. Pettersen *et al.*, UCSF Chimera--a visualization system for exploratory research and analysis. *Journal of computational chemistry* **25**, 1605-1612 (2004).
